# Integrative transcriptome and proteome revealed high-yielding mechanisms of epsilon-poly-L-lysine by *Streptomyces albulus*

**DOI:** 10.3389/fmicb.2023.1123050

**Published:** 2023-04-20

**Authors:** Liang Wang, Hao Yang, Mengping Wu, Jianhua Zhang, Hongjian Zhang, Zhonggui Mao, Xusheng Chen

**Affiliations:** The Key Laboratory of Industrial Biotechnology, Ministry of Education, School of Biotechnology, Jiangnan University, Wuxi, Jiangsu, China

**Keywords:** epsilon-poly-L-lysine, *Streptomyces albulus*, omics analyses, environmental stress, metabolism regulation mechanism

## Abstract

**Introduction:**

ε-poly-L-lysine (ε-PL) is a high value, widely used natural antimicrobial peptide additive for foods and cosmetic products that is mainly produced by *Streptomyces albulus*. In previous work, we developed the high-yield industrial strain *S. albulus* WG-608 through successive rounds of engineering.

**Methods:**

Here, we use integrated physiological, transcriptomic, and proteomics association analysis to resolve the complex mechanisms underlying high ε-PL production by comparing WG-608 with the progenitor strain M-Z18.

**Results:**

Our results show that key genes in the glycolysis, pentose phosphate pathway, glyoxylate pathway, oxidative phosphorylation, and L-lysine biosynthesis pathways are differentially upregulated in WG-608, while genes in the biosynthetic pathways for fatty acids, various branched amino acids, and secondary metabolite by-products are downregulated. This regulatory pattern results in the introduction of more carbon atoms into L-lysine biosynthesis and ε-PL production. In addition, significant changes in the regulation of DNA replication, transcription, and translation, two component systems, and quorum sensing may facilitate the adaptability to environmental pressure and the biosynthesis of ε-PL. Overexpression of *ppk* gene and addition of polyP_6_ further enhanced the ε-PL production.

**Discussion:**

This study enables comprehensive understanding of the biosynthetic mechanisms of ε-PL in *S. albulus* WG-608, while providing some genetic modification and fermentation strategies to further improve the ε-PL production.

## 1. Introduction

Epsilon-poly-lysine (ε-PL) is a naturally occurring homopoly(amino acid) consisting of 25–35 L-lysine residues that are structurally linked by α-carboxy and ε-amino groups. As a cationic antimicrobial peptide, ε-PL exhibits broad spectrum antimicrobial activity. In addition, ε-PL has numerous, commercially valuable characteristics such as its safety for human consumption, its biodegradability and solubility in water, which have led to its various applications in food, pharmaceuticals, and cosmetics (Wang Z. Y. et al., 2021; Wang L. et al., 2021). Currently, ε-PL has been approved as food preservative in many countries including Japan, South Korea, the United States, and China. However, as a value-added product, low production capacity has limited its commercial application in different industries.

Since the initial discovery of ɛ-PL-producing *Streptomyces* in 1977, numerous engineering strategies have helped to overcome the extremely low ɛ-PL production in the original wild-type strain. Historically, conventional methods were commonly used for screening high ε-PL producers (Hiraki et al., 1998; Wu et al., 2016; Wang et al., 2017). With advances in molecular biology, several studies have used genetic recombination to efficiently engineer strains for ɛ-PL production, most commonly by increasing the supply of its precursor, L-lysine (Hamano et al., 2007; Li et al., 2021), by activating or overexpressing ɛ-PL synthetase (Purev et al., 2020; Wang C. Y. et al., 2020; Wang A. X. et al., 2020), by suppressing biosynthesis of its antibiotic by-products (Yamanaka et al., 2019), or by increasing the availability of oxygen and nitrogen (Xu et al., 2015a; Xu J. Z. et al., 2018; Xu D. L. et al., 2018; Xu Y. R. et al., 2018). However, the regulation of ɛ-PL metabolism in *Streptomyces albulus* is highly complex, and determining which steps are rate-limiting steps in ɛ-PL biosynthesis poses a major challenge for directed evolution-based efforts to generate high-producing strains (Liu et al., 2019; Xiang et al., 2020; Wang Z. Y. et al., 2021; Wang L. et al., 2021).

‘Omics technologies’ have emerged as powerful tools for identifying relevant genes and metabolites that control the differential physiological traits between the mutant and wild-type strains, while providing new genetic modification strategies for further enhancement of the production of metabolites (Li and Liu, 2017). For example, transcriptomics analysis revealed that putrescine overproduction may be related to upregulated genes involved in ornithine biosynthesis and NADPH-biosynthetic enzymes in *Corynebacterium glutamicum* PUT-ALE. Subsequent CRISPRi-based suppression of NADPH- and ATP-consuming enzymes resulted in enhanced putrescine production in *C. glutamicum*. Recently, omics technologies were also utilized to resolve the molecular basis of high ɛ-PL production by *Streptomyces* (Liu et al., 2019) Metabolomics profiling revealed that the upregulation of glutamate, trehalose, and other metabolites related to L-lysine biosynthesis and degradation pathways might contribute to enhanced ɛ-PL production in high-producing mutants (Xiang et al., 2020; Wang Z. Y. et al., 2021; Wang L. et al., 2021). Moreover, comparative genomics of high-yield and low-yield strains identified potential genetic variants related to ɛ-PL yield (Wang et al., 2015a,b; Xiang et al., 2020). However, the conclusions available through individual ‘omics studies are limited to their respective cellular levels (i.e., protein, mRNA, small molecules, etc.), whereas integrative multi-omics analysis can provide more comprehensive perspective into the complex regulatory mechanisms between different layers of expression (Liu et al., 2020; Chen et al., 2021).

In our previous studies, we generated the ε-PL hyper-yielding strain *S. albulus* WG-608 through successive rounds of engineering the original strain M-Z18 by directed evolution (Li et al., 2012, 2013; Wang et al., 2015a,b; Wu et al., 2016). Here, we compare the physiological characteristics of WG-608 and M-Z18 cultured in a 5-L bioreactor using integrated transcriptomic and proteomics analyses. As a result of this combined analysis of whole cell metabolism in WG-608, in comparison with that in M-Z18, we gain a higher level of insight into the cellular landscape and factors that contribute to high ε-PL expression as well as related metabolic characteristics. To our knowledge, this work represents the first high resolution description of the molecular mechanisms underlying high ε-PL production in *S. albulus* through integrated physiological, transcriptomic, and proteomics analyses.

## 2. Materials and methods

### 2.1. Strain and medium

**Figure 1:**
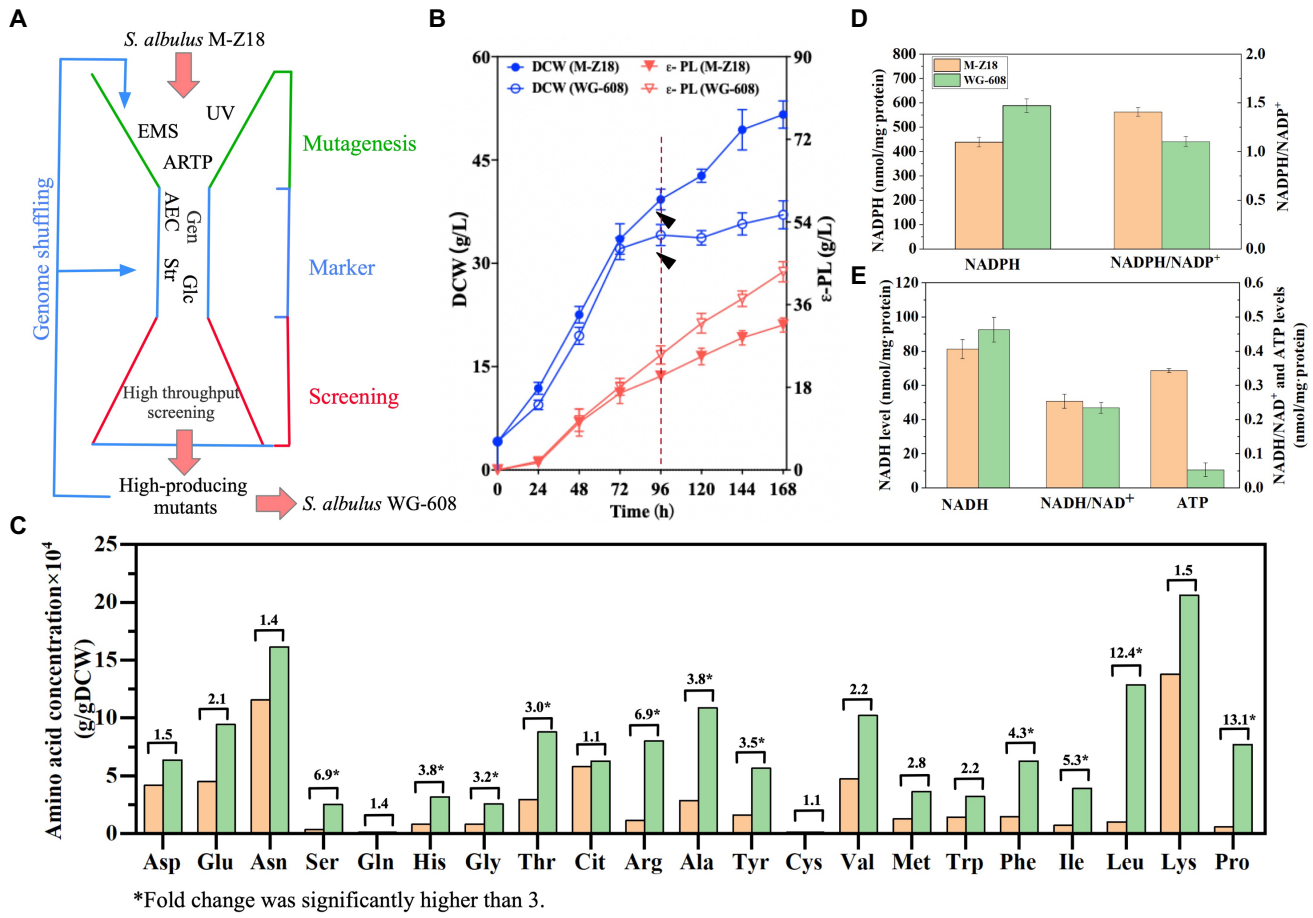
Comparison of the physiological characteristics between *S. albulus* M-Z18 and *S. albulus* WG-608. **(A)** Workflow for the *S. albulus* WG-608 bioengineering process. UV, ultraviolet mutagenesis; EMS, ethylmethylsulfone mutagenesis; ARTP, atmospheric and room temperature plasma; AEC, S-(2-aminoethyl)-L-cysteine; Str, Streptomycin; Gen, gentamycin; Glc; glucose; Asp., L-aspartate; Glu, L-glutamate; Asn, L-asparagine; Ser, L-serine; Gln, glutamine; His, L-histidine, Gly, L-glycine; Thr, L-threonine; Cit, Citrate; Arg, L-arginine; Ala, L-alanine; Tyr, L-tyrosine; Cys-s, L-cysteine; Val, L-valine; Met, L-methionine; Trp, L-tryptophan; Phe, L-phenylalanine; Ile, L-isoleucine; Leu, L-leucine; Lys, L-lysine; Pro, L-proline. **(B)** Dry cell weight and ε-PL production by *S. albulus* M-Z18 and *S. albulus* WG-608 in fed-batch fermentation. DCW, dry cell weight. **(C)** Intracellular amino acid levels at 96 h. Numbers above columns show fold difference and * indicates fold change was significantly higher than 3. **(D)** NADPH and NADPH/NADP^+^ levels at 96 h **(E)** NADH, NADH/NAD^+^ and ATP levels at 96 h.

*Streptomyces albulus* M-Z18 was utilized as a control strain achieved with ultraviolet mutagenesis of *S. albulus* Z-18 (CGMCC 10479). *S. albulus* WG-608 was a high-yielding mutant generate from *S. albulus* M-Z18 through genome shuffling, interspecific hybridization, ARTP mutagenesis and ribosome engineering (Figure 1) (Li et al., 2012, 2013; Wang et al., 2015a,b, 2017). *Escherichia coli* DH5α was used as cloning host, respectively. *E. coli* ET12567/pUZ8002 was used for *Streptomyces*—*E. coli* interspecies conjugation to introduce plasmids into *Streptomyces*. *E. coli* strains were all cultured at 37°C in Luria-Bertani medium (10 g/L tryptone, 5 g/L yeast powder, 10 g/L NaCl, pH 7.0) with the addition of final concentrations of 25 μg/mL kanamycin, 50 μg/mL apramycin, and 25 μg/mL chloramphenicol, if necessary. MS medium containing 20 g/L mannitol, 20 g/L soybean powder, 10 mM MgCl_2_, 20 g/L agar powder (pH 7.0) was used for intergeneric conjugation between *S. albulus* and *E. coli* at 30°C. Moreover, the final concentrations of 50 μg/mL apramycin and 25 μg/mL nalidixic acid were overlapped on MS medium for recombinant colonies screening. Agar slant medium (BTN) and the seed culture medium (M3G) were described previously (Chen et al., 2011). Fermentation medium was composed of (g/L): glucose 50, (NH_4_)_2_SO_4_·8, yeast extract 8, MgSO_4_·7H_2_O 2, KH_2_PO_4_ 2, FeSO_4_ 0.04 and ZnSO_4_ 0.04. Initial pH was adjusted to 6.8 by 2 M NaOH solution and/or 2 M H_2_SO_4_ before autoclaving. Glucose was autoclaved separately in each experimental group.

### 2.2. Plasmid and strain construction

To achieve the overexpression *ppk* in *S. albulus* WG-608, the method was performed as described by our previous study (Pan et al., 2019a,b) with some modifications. All primers used in this paper are located in Supplementary Table S1. The DNA fragment encoding *ppk* (Gene ID: 878853) was chemically synthesized (Aenta, Suzhou, China) with codon optimization. Under the catalysis of CloneExpress II One Step Cloning Kit (Vazyme, Nanjing, China), the obtained DNA fragments were ligated into the vector pIB139, respectively, which was digested by *Nde*I and *Eco*RI. Subsequently, ligation product was then transformed into competent *E. coli* DH5α, and exconjugants were picked out from LB plates containing 50 μg/mL apramycin. After validation by colony PCR using the primer pair C-F/-R and DNA sequencing (Aenta, Suzhou, China), the overexpression vectors pIB139-*ppk* was obtained.

Finally, the overexpression vector pIB139-*ppk* was separately transformed into *E. coli* ET12567 for intergeneric conjugation with *S. albulus* WG-608. To obtain overexpression strain OE-*ppk,* the transformants were screened on BTN solid medium supplemented with apramycin and nalidixic acid, and the colonies were verified by PCR using the primer pair P-F/-R.

### 2.3. Culture conditions

Slant and plate cultures: spores (2 × 10^8^–5 × 10^8^ CFU/mL) were inoculated on slant medium for 8–10 days at 30°C. Fermentation performance of *S. albulus* WG-608 and *S. albulus* M-Z18 were verified in a 5 L bioreactor (BIOTECH-5BG, BaoXing Bio-Engineering Equipment, China) with a working volume of 3.5 L. Seed culture was performed in a 500-mL Erlenmeyer flask with 80-mL M3G medium, incubating on a shaker for 24–30 h at 200 r/min. Approximately 240-mL pre-cultured seed was inoculated into the 5-L fermenter containing 3.26-L sterile YH fermentation medium at 30°C. During fermentation, pH naturally decreased and automatically maintained at 4.0 by pH electrodes. The concentrations of glucose and NH_3_-N were maintained at 1–10 g/L and 0.5–1.0 g/L, respectively, by automatically pulsed aseptic glucose solution (70%, w/v) and (NH_4_)_2_SO_4_ solution (600 g/L).

### 2.4. RNA sequencing and transcriptome analyses

For transcriptome analyses, 5 mL samples were separately taken from three independent fed-batch fermentation at 96 h. Then, these samples were mixed immediately and frozen in liquid nitrogen for the extraction of total RNA. Total RNA of WG-608 and M-Z18 samples were extracted using HiScript III RT SuperMix for qPCR(+gDNA wiper) (Vazyme, China). To reduce sequencing interference, DNA was removed using DNase I (NEB, United States), and rRNA was digested by RiboCop rRNA Depletion Kit for Gram Positive Bacteria (G^+^). The obtained RNA was then interrupted by RNA Fragmentation Buffer, and reverse-transcribed to synthesized double-strand cDNA by using a N6 randomized primer. The 5′ ends of the obtained double-stranded cDNA fragments were phosphorylated, while “A” bases were added to the 3′ ends for repairing the end of the DNA fragments. After that, the suitable ligation products were amplified by PCR using specific primers. The obtained PCR product was denatured by heat and formed single-stranded DNA, then single-stranded circular DNA library was constructed from the single-stranded DNA by circularization.

The DNA library was sequenced by Illumina HiSeqTM 2000, and the raw reads were obtained to determine whether the sequencing data is suitable for subsequent analysis. The obtained clean reads were filtered by evaluating statistical comparison rate and reads distribution on reference sequence. Then, the filtered clean reads were compared to the genome of *S. albulus* ZPM (NCBI accession no. NZ_CP006871) through SOAPaligner/SOAP2. Finally, differentially expressed genes (DEPs) with transcriptional differences more than 2 folds (FDR < 0.001, *p*-values <0.001) between the WG-608 and M-Z18 samples were selected under the conditions of FDR < 0.001 and p-values <0.001. Gene Ontology (GO)^1^ and Kyoto Encyclopedia of Genes and Genomes (KEGG)^2^ enrichment analysis were performed using GO and KEGG analysis Clusterprofiler software (Shen et al., 2022).

### 2.5. Protein extractions, LC–MS/MS analysis, and proteomic data processing

For protein extraction, 1 mL of cell samples were harvested from three separate replenished fractionated fermentations at 96 h. Bacteria were collected by centrifugation at 10,000 × g for 1 min at 4°C and the precipitate was washed three times with 1 mL of PBS buffer (pH 7.2) to remove material from the medium. Add an appropriate amount of 1XCocktai (containing SDSL3, EDTA) and glass beads. Place the mixture on ice for 5 min and add DTT at a final concentration of 10 mM. Subsequently, the organisms were lysed using a grinding device (60 W, 2 min) and then centrifuged (25,000 × g, 15 min) at 4°C to remove the supernatant. The supernatant was transferred to a clean centrifuge tube, then a final concentration of 10 mM DTT was added to a water bath at 56°C for 60 min. A final concentration of 55 mM IAM was added and stored away from light for 45 min. To precipitate the protein, pre-chilled acetone was added to the protein crude extract, mixed and kept at −20°C for 30 min. After resting, the proteins were collected by centrifugation (25,000 × g, 15 min) at 4°C. The proteins were freeze-dried and added to an appropriate amount of SDSL3. Subsequently, the proteins were lysed and centrifugated at 4°C (25,000 × g, 15 min) to remove the supernatant. Protein concentration in the protein solution was determined using TaKaRa Bradford Protein Assay Kit (TaKaRa, Japan).

For the enzymatic digestion of protein, 100 μg of protein solution was placed in a 1.5-mL centrifuge tube with 5-μg trypsin. Then the solution was vortex mixed, centrifugated at low speed for 1 min and incubate at 37°C for 2 h to digest the protein. The digested peptide solution was desalted using a HiTrap desalting column (GE Healthcare, United States) and the desalted peptide solution was freeze-dried. A 100 μg peptide mixture of each sample was labeled using iTRAQ reagent according to the manufacturer’s instructions (ThermoFisher, United States).

The samples were separated in liquid phase using a Shimadzu LC-20AB liquid phase system with a 5 μm 4.6 × 250 mm Gemini C18 column. The peptide samples were re-dissolved and drained using mobile phase A (5% ACN pH 9.8) and injected into the sample, eluting at a flow rate gradient of 1 mL/min. Condition of gradient elution were as follows: 5% mobile phase B (95% ACN, pH 9.8) for 10 min, 5% to 35% mobile phase B for 40 min, 35% to 95% mobile phase B for 1 min, mobile phase B for 3 min, and 5% mobile phase B equilibrated for 10 min. The elution peaks were monitored at 214 nm and the fraction was collected per minute. Twenty fractions were obtained by analyzing chromatographic elution peak maps and freeze drying. The dried peptide samples were re-dissolved with mobile phase A (2% ACN, 0.1% FA), centrifuged at 20,000 g for 10 min, and the supernatant was taken into the sample. Separation was performed by UltiMate 3,000 UHPLC (ThermoFisher, United States). Samples were first enriched and desalted in a trap column, followed by separation in tandem with a self-loading C18 column (AB SCIEX, United States) at a flow rate of 300 nL/min through the following effective gradient. Condition of gradient elution were as follows: 5% mobile phase B (98% ACN, 0.1% FA) for 5 min; mobile phase B increased linearly from 5% to 25% for 40 min; mobile phase B from 25% to 35% for 5 min; mobile phase B from 35% to 80% for 2 min; 80% mobile phase B for 2 min; 5% mobile phase B for 2 min.

After liquid-phase separation, the peptides were ionized by a nanoESl source and entered into a tandem mass spectrometer Orbitrap Fusion Lumos (ThermoFisher, United States) for DDA mode detection. The main parameters were set as follows: ion source voltage was set to >2 kV; primary mass spectrometry scan range 350–1,500 m/z; resolution was set to 60,000; secondary mass spectrometry starting point was fixed at 100 m/z; resolution was 15 km. The parent ions for secondary fragmentation were selected by: the top 30 parent ions with intensity of charge^2+^ to charge^6+^ peaks over 20,000. The ion fragmentation mode was HCD, and the fragment ions were detected in Orbitrap.

### 2.6. qRT-PCR validation

Mycelia of ε-PL hyper-yielding strain WG-608 and the original strain M-Z18 were sampled at 96 h during the fed-batch fermentation, then 10 DEGs (*sdhA*, *pls*, *metH*, *gltA*, *aceA*, *aspB*, *typB*, *glk*, *ppc*, *ppdk*) associated with ε-PL biosynthesis pathway were analyzed by qRT-PCR to ensure the reliability of RNA-sequencing. Total RNA obtained from same individuals was extracted using HiScript III RT SuperMix for qPCR(+gDNA wiper) (Vazyme, China). cDNA was synthesized using AMV First Strand cDNA Synthesis Kit (Sangon Biotech, China) based on the kit instructions, while the specific primers for target genes were designed using Beacon Designer 7 software and listed in Supplementary Table S2. The qRT-PCR experiment was performed as described by Du et al. (2022) using StepOne Real-Time PCR (Applied Biosystems, United States) and SYBRR Premix Ex TaqTM (Takara, Japan) with the following procedures: pre-incubation at 95°C for 30 s; 40 cycles at 95°C for 5 s, 60°C for 30 s, and cooling at 50°C for 30 s. The 20 μL reaction system was composed of 10.0 μL 2 × ChamQ Universal SYBR qPCR Master Mix, 2 μL DNA/cDNA template, 0.4 μL forward and reverse primers (10 μM), and 7.2 μL dH_2_O.The housekeeping gene *hrdB*, encoding RNA polymerase principal sigma factor, was selected as the reference gene for normalization.

### 2.7. Analytical methods

The fermentation broth in 5-L fermenter was sampled and centrifuged at 4,500 × g for 5 min. The obtained sediments were oven dried at 105°C for 12 h to measure the DCW. The supernatant was used to determine the concentrations of ε-PL, residual glucose and NH_4_^+^-N. ε-PL concentration was determined according to the description of Itzhaki (1972). Glucose concentration was detected by a biosensor analyzer (SBA-40D, Shandong Academy of Sciences, China), and NH_4_^+^-N was measured using Nessler reagent by the colorimetric method. The concentrations of intracellular NADPH and NADP^+^ were detected using NADP^+^/NADPH Assay Kit with WST-8 (Beyotime Biotech, China). ATP concentration was measured using ATP Assay Kit (Beyotime Biotech, China). Protein concentration was detected according to the instructions of Super-Bradford Protein Assay Kit (Takara, Japan). All above assays were performed in triplicate.

### 2.8. Statistical analysis

All experiments were conducted three times, and all data were expressed as mean ± standard deviation. The SPSS (version 22.0, SPSS Inc., Chicago, IL, United States) was used for statistical analysis that was performed using one-way analysis of variance (ANOVA) and Tukey’s test at *p* < 0.05.

## 3. Result and discussion

### 3.1. Changes of intracellular energy and amino acid levels in *Streptomyces albulus* WG-608

*Streptomyces albulus* WG-608 is a hyper-yielding mutant producer of ε-PL derived from *S. albulus* M-Z18 through multiple rounds of conventional mutagenesis, genome shuffling, and ribosome engineering (Figure 1A). In order to establish a mechanistic basis for enhanced ε-PL biosynthesis in WG-608, we first compared its intracellular energy and amino acid levels with that of M-Z18. During a 168 h fed-batch fermentation, WG-608 exhibited characteristically lower biomass production with higher ε-PL production compared to M-Z18 (Figure 1B). After roughly 96 h of cultivation in fed-batch fermentation, both strains entered stationary phase, after which WG-608 grew obviously slower than M-Z18, indicating greater downregulation of cell growth in WG-608. Despite the lower biomass, WG-608 generated significantly higher ε-PL biosynthesis, secreting 44.74 g/L ε-PL by 168 h of fermentation, 46.7% more than M-Z18.

The composition and concentration of intracellular amino acids were detected by HPLC (Figure 1C). L-lysine serve as the precursor for ε-PL biosynthesis, while L-aspartate and L-glutamate are primary precursors and amino donors for L-lysine biosynthesis (Wang C. Y. et al., 2020; Wang A. X. et al., 2020). Considering these cellular requirements, the relatively high intracellular concentrations of L-lysine, L-aspartate, and L-glutamate were highly conducive for ε-PL production. Moreover, all amino acid contents were increased to varying degrees in WG-608. The contents of L-threonine and L-methionine, two branched amino acids in the L-lysine biosynthesis pathway that also use L-aspartate as substrate, were also significantly increased (2.97- and 2.85-fold, respectively) in WG-608. Other aliphatic amino acids, such as L-proline, L-arginine, L-leucine and L-isoleucine, L-alanine and L-valine were increased by 2.2–13.1-fold that in M-Z18. These results indicated that WG-608 had considerably greater capability for essential amino acid biosynthesis. Consistent with our results, Wang Z. Y. et al. (2021) and Wang L. et al. (2021) also found that the synthesis of amino acids (i.e., L-lysine, L-threonine and L-proline) was upregulated in the high ε-PL-producing strain *Streptomyces diastatochromogenes* 6#-7 (Wang Z. Y. et al., 2021; Wang L. et al., 2021). Additionally, other studies have shown that L-arginine levels contribute to the arginine deaminase pathway to enhance acid resistance, while high L-glutamate and L-proline levels promote cellular tolerance and acclimation to environmental extremes.

NADPH is an essential component in L-lysine production because the synthesis of 1 mol of L-lysine from L-aspartate requires consumption 4 mol of NADPH (Xu J. Z. et al., 2018; Xu D. L. et al., 2018; Xu Y. R. et al., 2018). As shown in Figure 1D, WG-608 exhibited higher NADPH levels and a lower NADPH/NADP^+^ ratio than that of M-Z18, suggesting proportionally greater utilization of NADPH for L-lysine biosynthesis, accompanied by higher NADP^+^ generation. ATP also plays a vital role in ε-PL production mainly because the first step in conversion of L-lysine requires ATP-mediated activation of L-lysine monomers at the adenylation domain of ε-PL synthetase (Yamanaka et al., 2008). However, unexpectedly, intracellular ATP concentration was significantly lower in WG-608, although free NADH concentration and utilization were higher than that in M-Z18 (Figures 1D,E). These results are potentially attributable to excessive ATP consumption during the ε-PL biosynthesis once cells enter the stationary phase. Overall, the combined upregulation of amino acid metabolism and increased availability of NADPH and NADH in WG-608 appear to collectively enhance cellular homeostasis and ε-PL biosynthesis by WG-608.

### 3.2. Transcriptomics analysis

#### 3.2.1. Illumina HiSeq mRNA sequencing

In general, high-performance phenotypes can result from unique combinations of genomes (Yonemaru et al., 2014), but the effectiveness of these combinations requires successful integration regulatory mechanisms. Comparative transcriptomics can thus provide insight into the regulation of essential traits, such as high yields and response to adverse environmental conditions (Liu et al., 2020). We therefore carried out transcriptomic analysis of WG-608 and M-Z18 using the Illumina HiSeq 2000 platform to determine how critical changes in metabolism were controlled in WG-608. In total, we obtained 35.23 and 38.44 million clean reads from WG-608 and M-Z18, respectively, using SOAP2 alignment in conjunction with the NCBI database. Among them, 3,968 differential expressed genes (DEGs) were identified (*p* < 0.05), including 1965 up- and 2003 downregulated genes. GO analysis revealed that 940, 2,365 and 819 DEGs were enriched in cellular component, molecular function and biological process, respectively. The identified DEGs were mainly associated with the categories membrane part (797 DEGs) and membrane (813 DEGs) in cellular component, binding (1,204 DEGs) and catalytic activity (1,689 DEGs) in molecular function, as well as metabolic process (510 DEGs) and cell process (487 DEGs) in biological process (Supplementary Figure S1). KEGG analysis indicated that terms related to DNA replication and biotin metabolism were enriched with upregulated DEGs, while secondary metabolites and fatty acid biosynthesis were enriched with downregulated genes in WG-608 (Supplementary Figure S1).

#### 3.2.2. Transcriptional upregulation of DNA replication and repair, transcription and translation in WG-106

Based on the KEGG analysis showing preferential enrichment for DEGs in DNA replication and repair processes, we next focused on 17 highly significant DEGs in WG-608 identified by comparison with M-Z18 (Table 1), including 16 up- and 1 downregulated genes. The most highly upregulated DEGs included DNA polymerase I, DNA polymerase III subunits α, δ, ε and γ, two replicative DNA helicases, DNA-3-methyladenine glycosylase, and double-stranded uracil-DNA glycosylase, while only ribonuclease H1 expression was decreased. In addition, 14 DEGs annotated as RNA polymerase sigma factors were upregulated, implying the strong activation of RNA synthesis in WG-608 (Table 1). Gene expression of ribonuclease R, responsible for ribosomal RNA quality control and defective RNA degradation (Domingues et al., 2015), was upregulated by 14.3-fold compared to its expression in M-Z18, suggesting a heightened need for this function in WG-608. Furthermore, 7 DEGs related to protein synthesis were upregulated, including 2 ribosomal proteins (L14 and L17) and 2 tRNA ligases (lysine--tRNA ligase and cysteine--tRNA ligase), 1 methionyl-tRNA formyltransferase, and 1 elongation factor Tu, which together indicated a substantial increase in the rate and efficiency of translation. These results showing elevated regulation of DNA replication and repair, transcription, and translation likely contribute to modulating cell proliferation and homeostasis in WG-608, which may extend the life span, and hence productivity, of WG-608 cells.

**Table 1:** Classification of differential expression genes in replication and repair, transcription and translation.

	Gene name	Gene ID	Entry	Definition	log_2_ fold change (WG-608/M-Z18)
Replication and repair	*tag*	M-Z18AGL003258	K01246	DNA-3-methyladenine glycosylase I	1.23
	*ligD*	M-Z18AGL001247	K01971	Bifunctional non-homologousend joining protein ligD	1.74
	*dnaB*	M-Z18AGL004117	K02314	Replicative DNA helicase	3.29
	*dnaB*	M-Z18AGL004585	K02314	Replicative DNA helicase	2.01
	*polA*	M-Z18AGL003616	K02335	DNA polymerase I	3.35
	*polA*	M-Z18AGL004040	K02335	DNA polymerase I	1.12
	*dnaE*	M-Z18AGL006933	K02337	DNA polymerase III subunit alpha	5.62
	*holB*	M-Z18AGL004051	K02341	DNA polymerase III subunit delta	1.71
	*dnaQ*	M-Z18AGL006678	K02342	DNA polymerase III subunit epsilon	2.56
	*dnaX*	M-Z18AGL003741	K02343	DNA polymerase III subunit gamma/tau	4.99
	*mug*	M-Z18AGL001163	K03649	Double-stranded uracil-DNA glycosylase	1.63
	*uvrD*	M-Z18AGL003207	K03657	DNA helicase II/ATP-dependent DNA helicase PcrA	1.26
	*uvrA*	M-Z18AGL001527	K03701	Excinuclease ABC subunit A	1.19
	*LIG1*	M-Z18AGL006902	K10747	DNA ligase 1	1.4
	*ku*	M-Z18AGL001248	K10979	DNA end-binding protein Ku	1.26
	*ada-alkA*	M-Z18AGL001986	K13529	DNA-3-methyladenine glycosylase II	1.61
	*udg*	M-Z18AGL000913	K21929	Uracil-DNA glycosylase	−7.18
	*rnhA*	M-Z18AGL008343	K03469	Ribonuclease H1	−13.18
Transcription	*fliA*	M-Z18AGL002780	K02405	RNA polymerase sigma factor for flagellar operon FliA	2.09
	*rpoE*	M-Z18AGL003436	K03088	RNA polymerase sigma-70 factor, ECF subfamily (SigE)	1.99
	*rpoE*	M-Z18AGL007233	K03088	RNA polymerase sigma-70 factor, ECF subfamily (SigE)	−1.47
	*rpoE*	M-Z18AGL007663	K03088	RNA polymerase sigma-70 factor, ECF subfamily (SigE)	2.67
	*rpoE*	M-Z18AGL001305	K03088	RNA polymerase sigma-70 factor, ECF subfamily (SigE)	2.05
	*rpoE*	M-Z18AGL004676	K03088	RNA polymerase sigma-70 factor, ECF subfamily (SigE)	3.14
	*rpoE*	M-Z18AGL005816	K03088	RNA polymerase sigma-70 factor, ECF subfamily (SigE)	2.18
	*rpoE*	M-Z18AGL003390	K03088	RNA polymerase sigma-70 factor, ECF subfamily (SigE)	1.37
	*rpoE*	M-Z18AGL004914	K03088	RNA polymerase sigma-70 factor, ECF subfamily (SigE)	3.61
	*rpoE*	M-Z18AGL004260	K03088	RNA polymerase sigma-70 factor, ECF subfamily (SigE)	−1.26
	*rpoE*	M-Z18AGL004677	K03088	RNA polymerase sigma-70 factor, ECF subfamily (SigE)	2.72
	*rpoE*	M-Z18AGL005143	K03088	RNA polymerase sigma-70 factor, ECF subfamily (SigE)	4.9
	*rpoE*	M-Z18AGL005709	K03088	RNA polymerase sigma-70 factor, ECF subfamily (SigE)	1.15
	*rpoE*	M-Z18AGL003653	K03088	RNA polymerase sigma-70 factor, ECF subfamily (SigE)	1.4
	*sigB*	M-Z18AGL004215	K03090	RNA polymerase sigma B factor	2.02
	*sigB*	M-Z18AGL004216	K03090	RNA polymerase sigma B factor	1.15
	*sigB*	M-Z18AGL005154	K03090	RNA polymerase sigma B factor	−2.97
	*vacB*	M-Z18AGL001852	K12573	Ribonuclease R	−3.84
Translation	*lysS*	M-Z18AGL001476	K04567	Lysine-tRNA ligase	1.3
	*cysS*	M-Z18AGL003796	K01883	Cysteine-tRNA ligase	1.24
	*rpmG*	M-Z18AGL004814	K02913	Large subunit ribosomal protein L33	−1.1
	*rplN*	M-Z18AGL004845	K02874	50S Ribosomal protein L14	1.02
	*rplQ*	M-Z18AGL004864	K02879	50S Ribosomal protein L17	1.07
	*fmt*	M-Z18AGL007471	K00604	Methionyl-tRNA formyltransferase	1.18
	*tuf*	M-Z18AGL001211	K02358	Elongation factor Tu	5.98

### 3.3. Proteomics analysis

#### 3.3.1. Protein identification and annotation

In order to further identify global changes in metabolism underlying enhanced ε-PL biosynthesis between WG-608 and M-Z18, we conducted comparative proteomics analysis using an iTRAQ technique. A total of 3,064 differentially expressed proteins (DEPs), including 1,473 upregulated and 1,591 downregulated proteins, were identified in WG-608. GO enrichment analysis suggested that these DEPs were mainly associated with the integral component of membrane (432 DEPs), cofactor binding (284 DEPs), and catalytic activity (1,532 DEPs), as well as response to stimulus (96 DEPs) and localization (107 DEPs) (Supplementary Figure S2). KEGG analysis suggested that upregulated proteins were primarily enriched in pathways related to oxidative phosphorylation, two-component system, and quorum sensing system, while downregulated proteins were enriched in valine, leucine, and isoleucine biosynthesis, as well as fatty acid metabolism (Supplementary Figure S2). However, we noted that KEGG enrichment analyses of the transcriptome and proteome showed relatively low overlap in enriched pathways, suggesting complexity in the regulatory mechanisms controlling gene and protein expression required for ε-PL production.

#### 3.3.2. High abundance of regulatory proteins in WG-608

In light of KEGG and GO enrichment analyses that showed significant changes in the primary and secondary metabolism of *S. albulus* after successive rounds of engineering, we next investigated DEPs related to signal transduction systems to better understand how WG-608 adapted to extracellular stimuli such as high ε-PL concentration and low pH, while continuing to secrete copious levels of ε-PL. In particular, we focused on two-component systems and quorum sensing due to their roles as signal transduction systems that mediate environmental sensing by membrane-associated sensor kinases and subsequent adaptation by response regulators of downstream target gene expression (Rapun-Araiz et al., 2020).

As shown in Supplementary Table S3, the MtrAB two-component system was upregulated in WG-608, including 3 MtrA and 7 MtrB isozymes. As reported in *Mycobacterium tuberculosis*, phosphorylated MtrA can promote DNA replication by binding to the promoter region of *dnaA* (Fol et al., 2006). In *Streptomyces coelicolor* A3(2) and *Streptomyces venezuelae*, MtrA is a key regulator of antibiotic production, and MtrA activation can lead to overproduction of chloramphenicol and actinorhodin (Som et al., 2017). Wang C. Y. et al. (2020) and Wang A. X. et al. (2020) found that the MtrAB upregulation in *S. albulus* should be appeared to be positively correlated with ε-PL biosynthesis. Hence, we speculated that increased MtrAB expression in WG-608 could promote enhanced DNA replication and ε-PL production. Additionally, the RegX3-SenX3 two-component system and alkaline phosphatase D were also found to be upregulated in WG-608. Alkaline phosphatase D is reportedly only activated under phosphate starvation conditions, and results in pro-survival responses, such as antibiotic synthesis or changes in respiration/energy production pathways, while the RegX3-SenX3 system is required for phosphate uptake and aerobic respiration (Marahiel et al., 1987; Sun et al., 1996; Rapun-Araiz et al., 2020). Consequently, phosphate transport system substrate-binding protein (PtsS) and the proteins related to oxidative phosphorylation were observed to be upregulated in WG-608 (Supplementary Table S3). These results implied that the phosphate supply could be insufficient during the late logarithmic phase (96 h) of fermentation in WG-608, resulting in alkaline phosphatase D and RegX3-SenX3 activation to upregulate PtsS and oxidative phosphorylation, ultimately increasing phosphate uptake and ATP generation for cell survival and ε-PL production.

MprAB and PepD belong to a complex signal transduction system that responds to cell envelope-related stress. In this signal cascade, the histidine kinase sensor MprB auto-phosphorylates under stress and transduces the stress signal to MprA and PepD, which together regulate the expression of stress-responsive factors, including sigE and sigB (Zahrt et al., 2003). Our previous results showing upregulation of MprA, sigE and sigB (Table 1 and Supplementary Table S1) were thus in agreement with the above studies (Parish, 2014). Remarkably, Pan et al. (2019a,b) found that high protein levels of MprAB and PepD during stress response to acidic conditions lead to upregulation of the ε-PL synthetase gene, while the deletion of MprA or MprB results in a ~50% decrease in its production.

In addition, we found that quorum-sensing systems regulated by cell density are also activated in WG-608. For instance, DegU, DegS, PleC, phospholipase C, and eukaryotic-like serine/threonine-protein kinase were significantly upregulated. Previous studies have shown that their function is predominantly associated with virulence, biofilm formation, and chemotaxis (Wright and Ulijasz, 2014; Kim, 2015; Fiester et al., 2016). We also observed that 11 DEPs associated with protein secretion and export were upregulated, including 5 preprotein translocase subunits (SecD, SecF, SecG, YajC, and SecY), 2 isozymes of signal peptidase I, 2 s-independent protein translocase proteins (TatB and TatC), and 2 YidC/Oxa1 insertase family membrane proteins. The upregulation of these enzymes thus likely drives increased extracellular secretion of auto-inducer oligopeptides, which could activate quorum-sensing responses by WG-608, despite the significantly lower cell concentrations than that of M-Z18. Interestingly, some transcription factors belonging to PadR and TetR/AcrR families were downregulated in WG-608. As reported, TetR-type regulators are negative regulators of lincomycin biosynthesis in *Streptomyces lincolnensis* (Xu Y. R. et al., 2018), and hence downregulation of TetR-type regulators may contribute to increased ε-PL production.

Moreover, protein abundance of VanS and VanJ, related to vancomycin resistance, was increased by 2.06- and 2.74-folds, respectively, potentially enhancing WG-608 tolerance to ε-PL. Three DEPs involved in toxoflavin biosynthesis (ToxC, ToxD and ToxA) and 1 DEP in anthranilate biosynthesis (TrpE) were all significantly lower in WG-608, possibly reducing the potential damage related to production of these agents (Abe et al., 2019). We also found that substrate-binding protein (ABC.SP.S) and ATP-binding protein (ABC.SP.A) were upregulated in WG-608. Their contribution to transport of spermidine and putrescine could limit their accumulation to toxic levels (Petronella and Ronholm, 2018) in *S. albulus.* Similarly, D-galactosaminyltransferase, a putative drug exporter in the RND superfamily, was upregulated by 2.02-fold. Additionally, several ATP-binding cassette transporters (ABC transporters) that participate in the transport of peptides, nickel, antibiotics, and branched-chain amino acids were also significantly upregulated in WG-608 (Supplementary Table S4). Glutamate decarboxylase was also upregulated by 1.28-fold, suggesting enhanced acid resistance in WG-608 (Wang C. Y. et al., 2020; Wang A. X. et al., 2020).

Taken together, proteomics analysis suggests that WG-608 adapts to diverse external conditions *via* activation of its quorum sensing and two-component regulatory systems, which promote cell proliferation, nutrient (phosphate and amino acids) uptake, energy supply, resistance to toxic metabolites (vancomycin, spermidine, and putrescine), tolerance to acid pH, and regulation high ε-PL synthesis.

### 3.4. Integrative analyses of transcriptome and proteome for ε-PL production

#### 3.4.1. Correlation between transcripts and proteins

**Figure 2:**
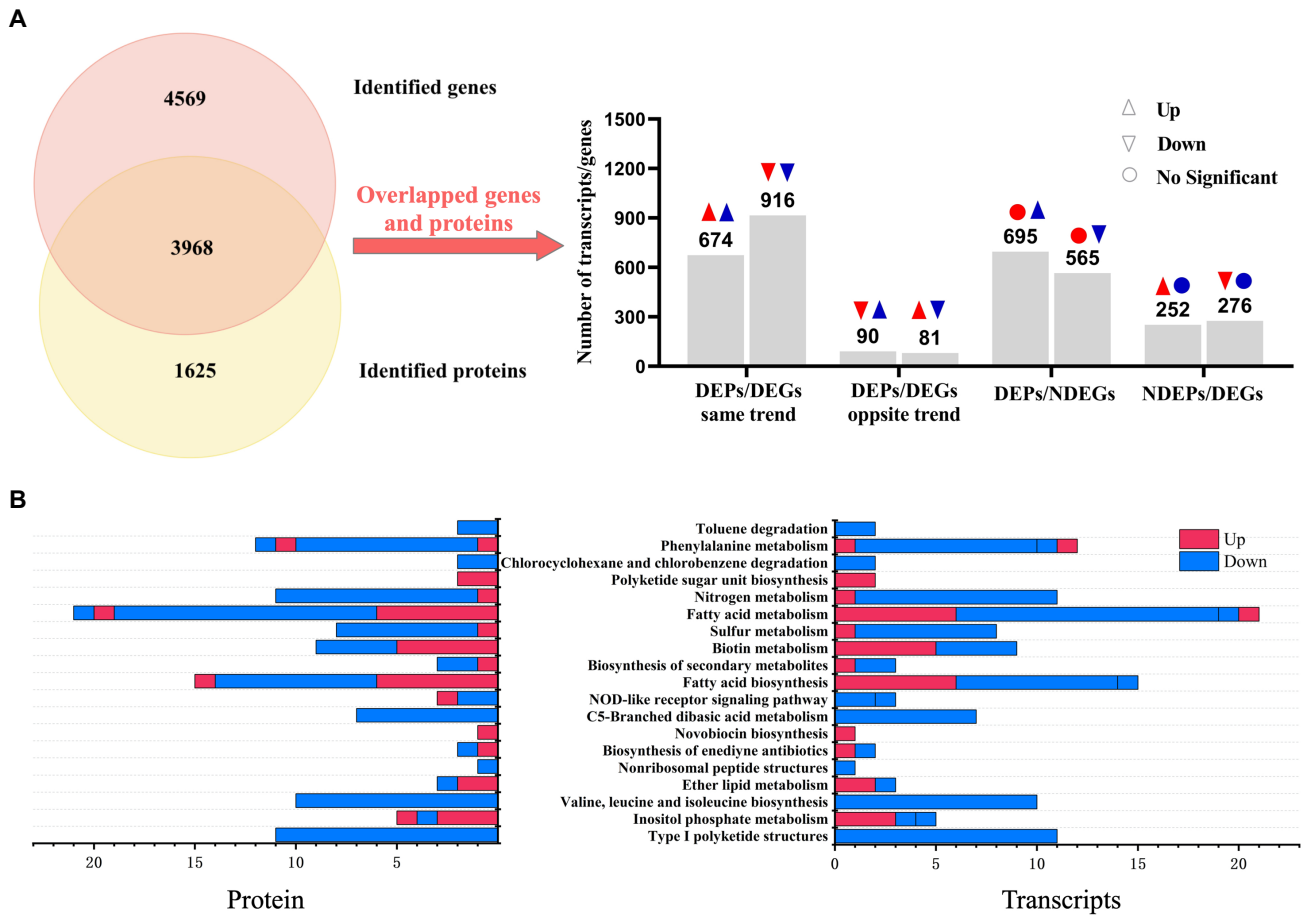
Association analysis of genes and proteins between the high ε-PL-producing mutant *S. albulus* WG-608 and its progenitor strain *S. albulus* M-Z18. **(A)** Numbers of overlapping gene/protein pairs that show the same or opposite trends in expression, or that show differential expression only at the protein or mRNA level between WG-608 and M-Z18. The red indicates transcript, blue indicates protein. **(B)** KEGG enrichment analysis of overlapping gene/protein pairs between *S. albulus* M-Z18 and *S. albulus* WG-608. Red represents pathways enriched with upregulated genes/proteins; blue represents pathways enriched with downregulated genes/proteins.

In order to better understand how regulation changed at the transcriptional or post-transcriptional levels in the development of WG608, we identified correlations between the differential expression patterns of genes (DEGs) and proteins (i.e., DEPs) from our above comparison of WG608 and M-Z18. A total of 3,968 DEGs overlapped with DEPs, of which 40% (1,590/3968) showed the same trend in upregulation or downregulation as the corresponding DEP, while 4.3% (171/3968) of DEGs showed the opposite trend of its corresponding DEP (Figure 2A). Additionally, 760 DEGs showed no change in protein level, whereas 528 DEPs showed no obvious difference in mRNA expression between strains (Figure 2A). To identify the metabolic pathways that were similarly regulated in WG-608 at the nucleotide and protein levels, we conducted KEGG enrichment analysis of these overlapping DEP/DEG pairs. KEGG annotation classified these DEPs and DEGs into 19 pathways (Figure 2B). Among these pathways, downregulated gene/protein pairs in WG608 were significantly enriched in type I polyketide structures, and in valine, leucine and isoleucine biosynthesis. By contrast, upregulated gene/protein pairs were associated with ether lipid metabolism. However, gene/protein pairs that showed the opposite trend in regulation were enriched pathways related to inositol phosphate metabolism and NOD-like receptor signaling.

**Figure 3:**
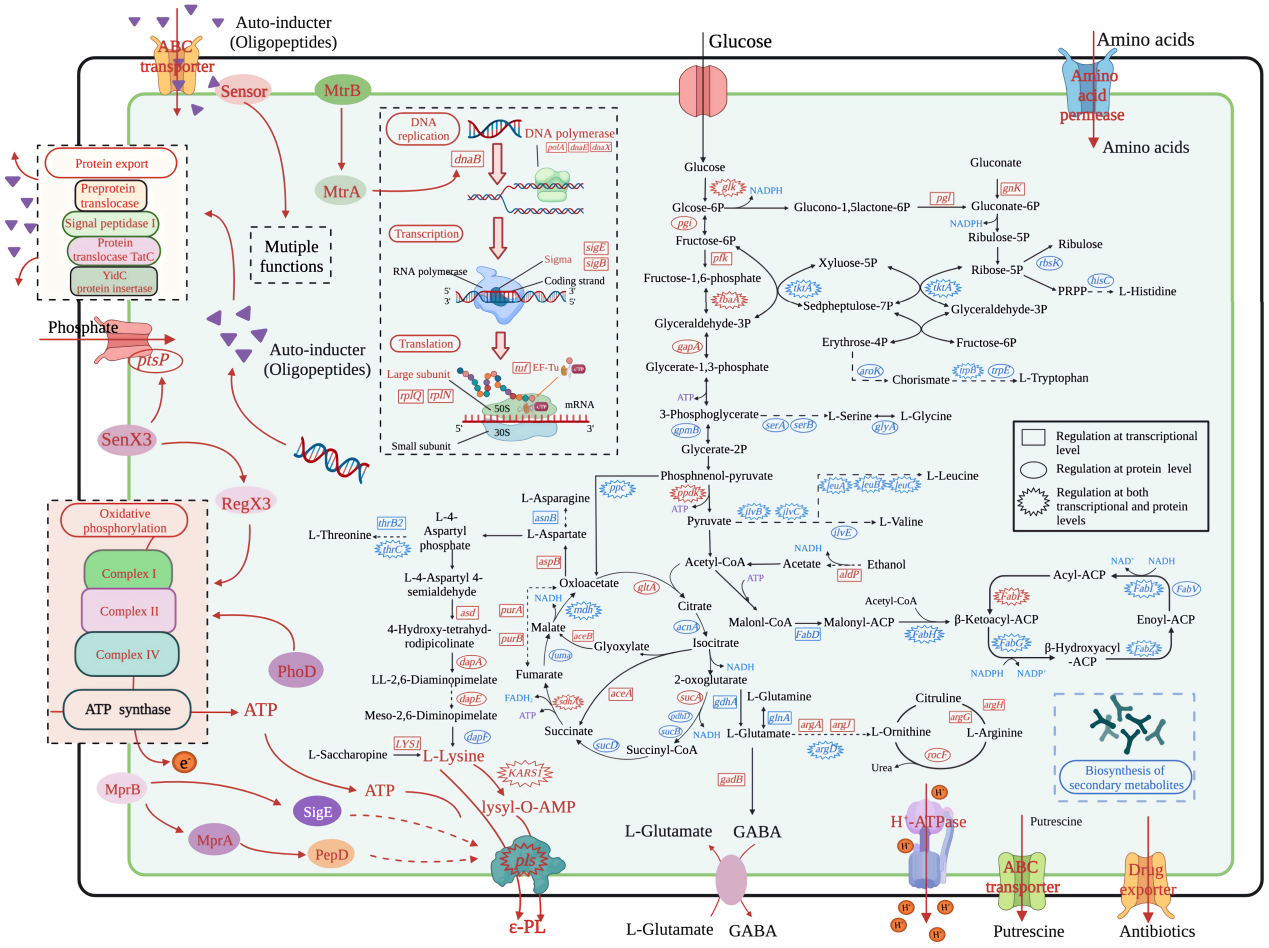
Overview of the transcriptional and translational regulation in the high ε-PL producer *S. albulus* WG-608. Red represents transcriptional upregulation/increased protein accumulation, whereas blue represents downregulation/decreased protein levels.

It is well known that ε-PL is formed by polymerization of L-lysine *via* ε-PL synthetase. In *S. albulus*, the carbon skeletons for L-lysine are mostly derived from the glycolysis pathway, pentose phosphate pathway (PPP), tricarboxylic acid (TCA) cycle, anaplerotic reactions, and the lysine biosynthesis pathway. Thus, other branched amino acids are considered important by-products of ε-PL biosynthesis. In addition, acetyl-CoA, the major substrate for fatty acid biosynthesis, is also an essential intermediate metabolite in L-lysine biosynthesis. We therefore focused on these pathways using integrated analysis of the transcriptomic and proteomics profiles to identify the molecular mechanisms responsible for the high ε-PL production in WG-608 (Figure 3).

#### 3.4.2. Glycolysis pathway

Our results showed that five proteins involved in the glycolysis pathway, including glucokinase (*glk*), fructose-bisphosphate aldolase (*pgi*), triosephosphate isomerase (*fbaA*), glyceraldehyde 3-phosphate dehydrogenase (*gapA*), and pyruvate, orthophosphate dikinase (*ppdk*) were all significantly upregulated, while we detected significant increases in transcripts for *glk*, glucokinase-6-phospho-beta-glucosidase (*celF*), *pgi*, ATP-dependent phosphofructokinase (*pfk*), *fbaA*, and *ppdk*. We also found that a putative phosphoglycerate mutase (*gpmB*) was downregulated at the protein level, although there was no difference in its mRNA expression between strains. As the first step and a key rate-limiting enzyme in the glycolysis pathway, glucokinase determines the volume of total carbon flux. The transcription and protein accumulation of glucokinase were both upregulated by 4.28- and 1.36-fold, respectively, indicating enhanced efficiency of glucose uptake and utilization in WG-608. Enhancement of the glycolysis pathway can promote the conversion and formation of important precursors in various metabolic pathways, such as pyruvate and acetyl-CoA in the TCA cycle or glucose-6-phosphate in the PPP. We therefore examined the regulation of these pathways to check for potential effects of enhanced glycolysis.

#### 3.4.3. Pentose phosphate pathway

The PPP is a primary source of NADPH, supplying the majority of intracellular NADPH in *S. albulus* through the activity of glucose 6-phosphate dehydrogenase (*zwf*) and 6-phosphategluconate (*gntZ*). Unexpectedly, we found no obvious changes in transcription or protein expression of these two enzymes between WG-608 and M-Z18. However, we did find significant differential upregulation of 6-phosphogluconolactonase (*pgl*) and gluconokinase (*gnk*) in WG-608, which could provide more gluconate-6phosphate substrate for 6-phosphategluconate to synthesize ribulose-5P. As a result, the PPP is strengthened, and more NADPH is generated, which was consistent with the increased intracellular NADPH concentration observed in WG-608 (Figure 1D). Additionally, the downregulation of ribokinase (*rbsK*) led to the shunting of carbon towards 5-phosphoribosyl 1-pyrophosphate (PRPP) synthesis, which is a critical intermediate in *de novo* nucleotide synthesis (Zhang et al., 2018). Notably, gene and protein expression levels of transketolase (*tktA*) were both significantly downregulated by 4.67- and 4.0-folds in WG-608, leading to reduced accumulation of sedoheptulose-7P and erythrose 4-phosphate, which in turn limits biosynthesis of the chorismate branch.

#### 3.4.4. TCA and glyoxylate cycles

The TCA cycle represents the primary hub for carbon skeletons essential for energy production and metabolism, which provides energy and precursors for carbohydrate, lipid, and amino acid synthesis. Citrate synthase (*gltA*) catalyzes the first and rate-limiting step in the TCA cycle, in which oxaloacetate and acetyl-CoA undergo condensation to form citrate. As shown in Table 2, citrate synthase transcription and protein were both strongly upregulated, suggesting that carbon flux was redirected from glycolysis to the TCA cycle in WG-608. However, compared to M-Z18, several other TCA cycle enzymes were slightly downregulated in WG-608 at the protein level, including aconitate hydratase (*acnA*), 2-oxoglutarate dehydrogenase (*sucA*, *sucB*, *pdhD*), succinyl-CoA synthetase alpha subunit (*sucD*), fumarate hydratase (*fumA*), and malate dehydrogenase (*mdh*). The downregulation of these enzymes could partially explain the limited cell growth of WG-608. However, decreased expression of TCA cycle enzymes appears beneficial to L-lysine production since it can lead to elevated accumulation of oxaloacetate, a precursor of L-lysine (Van Ooyen et al., 2012; Ainelo et al., 2019). For instance, Becker et al. (2009) reduced the carbon flux into the TCA cycle by inhibiting isocitrate dehydrogenase activity, resulting in increased L-lysine production (>40%) in *C. glutamicum*.

**Table 2:** Differential expression genes and proteins associated with carbohydrate metabolism and L-lysine biosynthesis between *S. albulus* WG-608 and *S. albulus* M-Z18.

Metabolic pathways	Gene name	Gene ID	Entry	Definition	log_2_ fold change (gene)	Fold change (protein)
Glycolysis	*glk*	M-Z18AGL001165	K00845	Glucokinase	2.10	1.36
	*celF*	M-Z18AGL005622	K01222	6-Phospho-beta-glucosidase	1.66	
	*pgi*	M-Z18AGL001662	K01624	Fructose-bisphosphate aldolase, class II	3.62	1.72
	*pfk*	M-Z18AGL006038	K21071	ATP-dependent phosphofructokinase/diphosphate-dependent phosphofructokinase	1.60	
	*fbaA*	M-Z18AGL001535	K01803	Triosephosphate isomerase (TIM)	3.61	1.69
	*gpmB*	M-Z18AGL008361	K15634	Probable phosphoglycerate mutase		−3.57
	*gapA*	tr|A0A1A9QTW9|A0A1A9QTW9_STRA9	K00134	Glyceraldehyde 3-phosphate dehydrogenase		1.49
	*poxB*	M-Z18AGL002314	K00156	Pyruvate dehydrogenase (Quinone)	2.79	
	*acs*	M-Z18AGL005013	K01895	Acetyl-CoA synthetase	3.27	
	*ppdk*	M-Z18AGL005661	K01006	Pyruvate, orthophosphate dikinase	2.79	1.45
	*ppc*	M-Z18AGL005090	K01595	Phosphoenolpyruvate carboxylase	−1.23	−1.56
TCA cycle	*pdhD*	M-Z18AGL003419	K00382	Dihydrolipoamide dehydrogenase		−1.32
	*pdhD*	M-Z18AGL007849	K00382	Dihydrolipoamide dehydrogenase	−9.75	−1.75
	*sucA*	tr|X0MVH2|X0MVH2_STRA9	K00164	2-Oxoglutarate dehydrogenase E1 component		1.45
	*sucB*	tr|A0A1A9QLF7|A0A1A9QLF7_STRA9	K00658	2-Oxoglutarate dehydrogenase E2 component (dihydrolipoamide succinyltransferase)		−1.39
	*sucB*	M-Z18AGL005977	K00658	2-Oxoglutarate dehydrogenase E3 component (dihydrolipoamide succinyltransferase)		−1.27
	*aceA*	M-Z18AGL007682	K01637	Isocitrate lyase	1.72	
	*aceB*	M-Z18AGL007679	K01638	Malate synthase	1.05	−1.25
	*fumA*	M-Z18AGL003320	K01676	Fumarate hydratase, class I		−1.52
	*fumA*	SG-86AGL005407	K01676	Fumarate hydratase, class I		−1.30
	*gyaR*	M-Z18AGL002895	K00015	Glyoxylate reductase		−1.75
	*korB*	M-Z18AGL004790	K00175	2-Oxoglutarate	−1.00	
	*purA*	M-Z18AGL001734	K01939	Adenylosuccinate synthase	1.35	
	*purA*	M-Z18AGL004630	K01939	Adenylosuccinate synthase	1.64	
	*purB*	M-Z18AGL001162	K01756	Adenylosuccinate lyase	1.58	
	*gltA*	M-Z18AGL003673	K01647	Citrate synthase	1.67	1.27
	*gltA*	M-Z18AGL005458	K01647	Citrate synthase		1.20
	*acnA*	WP_038522317.1	K01681	Aconitate hydratase		−1.41
	*adhc*	M-Z18AGL004911	K00121	Alcohol dehydrogenase	1.46	
	*mdh*	M-Z18AGL003522	K00024	Malate dehydrogenase	−2.05	−1.37
	*sdhA*	tr|A0A059WCE4|A0A059WCE4_STRA9	K00241	Succinate dehydrogenase/fumarate reductase, cytochrome b subunit		1.25
	*sdhA*	tr|X0MQF3|X0MQF3_STRA9	K00241	Succinate dehydrogenase/fumarate reductase, cytochrome b subunit		2.87
	*sdhA*	M-Z18AGL003296	K00240	Succinate dehydrogenase/fumarate reductase, iron–sulfur subunit	1.02	1.59
	*sdhA*	M-Z18AGL006117	K00239	Succinate dehydrogenase/fumarate reductase, flavoprotein subunit	1.98	1.57
	*sucD*	M-Z18AGL003533	K01902	Succinyl-CoA synthetase alpha subunit		−1.64
	*sucD*	M-Z18AGL003534	K01903	Succinyl-CoA synthetase beta subunit		−1.49
Pentose phosphate pathway	*pgl*	M-Z18AGL001545	K07404	6-Phosphogluconolactonase		1.44
	*pgl*	M-Z18AGL004720	K07404	6-Phosphogluconolactonase		
	*gntK*	M-Z18AGL003471	K00851	Gluconokinase	2.63	
	*tktA*	WP_038516370.1	K00615	Transketolase		−3.85
	*tktA*	M-Z18AGL001352	K00615	Transketolase	−4.67	
	*rbsK*	M-Z18AGL008413	K00852	Ribokinase		−4.00
Oxidative phosphorylation	*nuoA*	M-Z18AGL004812	K01485	Cytosine/Creatinine deaminase		−1.28
	*nuoH*	M-Z18AGL004774	K00337	NADH-quinone oxidoreductase subunit H		1.28
	*nuoN*	SG-86AGL003974	K00343	NADH-quinone oxidoreductase subunit N		1.47
	*nuoI*	WP_038520155.1	K00338	NADH-quinone oxidoreductase subunit I		1.20
	*nuoL*	M-Z18AGL004778	K00341	NADH-quinone oxidoreductase subunit L		1.35
	*nuoM*	M-Z18AGL004779	K00342	NADH-quinone oxidoreductase subunit M		1.54
	*nuoF*	M-Z18AGL004772	K00335	NADH-quinone oxidoreductase subunit F		1.25
	*nuoB*	M-Z18AGL004800	K00331	NADH-quinone oxidoreductase subunit B		−1.61
	*nuoG*	WP_038520158.1	K00336	NADH-quinone oxidoreductase subunit G		1.21
	*coxC*	M-Z18AGL006010	K02276	Cytochrome C oxidase subunit III		1.42
	*coxA*	M-Z18AGL006006	K02274	Cytochrome C oxidase subunit I		1.53
	*coxA*	WP_038519054.1	K02274	Cytochrome C oxidase subunit I		1.49
	*coxB*	M-Z18AGL006005	K02275	Cytochrome C oxidase subunit II		1.59
	*gph*	SG-86AGL000763	K01091	Phosphoglycolate phosphatase		1.87
	*ppa*	WP_038523209.1	K01507	Inorganic pyrophosphatase		1.36
	*ppk1*	M-Z18AGL003832	K00937	Polyphosphate kinase	2.19	1.54
DAP	*aspB*	M-Z18AGL001941	K00812	Aspartate aminotransferase	1.13	
	*asd*	M-Z18AGL005529	K00133	Aspartate-semialdehyde dehydrogenase	1.44	
	*dapA*	M-Z18AGL005888	K01714	4-Hydroxy-tetrahydrodipicolinate synthase		1.64
		M-Z18AGL007081	K01714	4-Hydroxy-tetrahydrodipicolinate synthase		1.63
	*dapB*	M-Z18AGL002689	K00215	4-Hydroxy-tetrahydrodipicolinate reductase		−1.2
	*dapD*	M-Z18AGL006255	K00674	2,3,4,5-Tetrahydropyridine-2,6-dicarboxylate n-succinyltransferase		−1.22
	*dapE*	M-Z18AGL003206	K01436	Amidohydrolase		1.34
	*dapF*	M-Z18AGL002630	K01778	Diaminopimelate epimerase		−1.45
	*purA*	M-Z18AGL001734	K01939	Adenylosuccinate synthase	1.35	
	*purA*	M-Z18AGL004630	K01939	Adenylosuccinate synthase	1.64	
	*purB*	M-Z18AGL001162	K01756	Adenylosuccinate lyase	1.58	
	*murE*	M-Z18AGL001071	K01928	UDP-N-acetylmuramoyl-L-alanyl-D-glutamate--2,6-diaminopimelate ligase		1.43
	*lys1*	M-Z18AGL001656	K00290	Saccharopine dehydrogenase (NAD^+^, L-lysine forming)	3.53	

Interestingly, we found that the majority of enzymes involved in the glyoxylate cycle were upregulated (Table 2). The glyoxylate cycle serves as a shunt or short alternative to the TCA cycle, in which the decarboxylation steps are bypassed by reactions catalyzed by isocitrate lyase (*aceA*) and malate synthase (*aceB*) (Acetyl-CoA + NAD^+^ → Succinate + NADH + CoA) (Chew et al., 2021). Our transcriptomic data showed that *aceA* and *aceB* mRNAs were differentially upregulated by 3.29- and 2.08-fold, respectively. Four DEGs and two DEPs annotated as succinate dehydrogenase (*sdhA*), an enzyme shared by the TCA and glyoxylate cycles, were all significantly upregulated. It is reasonable to hypothesize that enhanced glyoxylate cycle could cause increased the efficiency of four-carbon compound synthesis (such as succinate and malate) from acetyl-CoA, and higher accumulation of oxaloacetate for L-lysine production.

#### 3.4.5. Anaplerotic reactions

Anaplerotic reactions represent another important pathway through which oxaloacetate used by phosphoenolpyruvate carboxylase (*ppc*) (PEP + CO_2_ + ADP → OAA) can be replenished. However, *ppc* protein and gene expression were downregulated by 1.56- and 2.34-fold, respectively. This result was unexpected because recent studies have shown that elevated phosphoenolpyruvate carboxylase activity benefits ε-PL biosynthesis (Xu et al., 2015b; Wang Z. Y. et al., 2021; Wang L. et al., 2021). We therefore speculated that the downregulation of phosphoenolpyruvate carboxylase could be part of a response to the substantial accumulation of OAA resulting from upregulation of the glyoxylate cycle. This possibility further supports *ppc* as a strong candidate gene for engineering ε-PL production.

#### 3.4.6. ATP supply

As a pivotal molecule for energy transfer in the vast majority of cellular processes, ATP is mainly generated by glycolysis, the TCA cycle, and oxidative phosphorylation. Transcription of alcohol dehydrogenase (*adhc*), an essential enzyme in ATP synthesis, was upregulated 2.76-fold in WG608 (Table 2). This finding was consistent with a slight increase in WG-608 NADH levels (Figure 1E), since the reaction catalyzed by alcohol dehydrogenase is accompanied by NADH generation. Pyruvate phosphate dikinase (*ppdk*) and polyphosphate kinase (*ppk1*) mRNA and protein were also both upregulated, which could facilitate ATP generation. In addition, we detected enhanced expression of enzymes involved in oxidative phosphorylation, including NADH-quinone oxidoreductase, cytochrome c oxidase, F-type H^+^-transporting ATPase, phosphoglycolate phosphatase, inorganic pyrophosphatase. Collectively, these results would suggest a substantially greater ATP supply in WG-608 than M-Z18, but in fact, WG-608 had lower intracellular ATP levels than M-Z18. This effect could be due to high ATP consumption in the synthesis of ε-PL, which led us to next scrutinize the regulation of genes and proteins required for L-lysine production. We also noted that further metabolic engineering to strengthen ATP synthesis could be a tractable means of improving ε-PL production.

#### 3.4.7. L-lysine biosynthesis

In *S. albulus*, L-lysine is synthesized from L-aspartate through the succinyl-diaminopimelic acid pathway. In this pathway, aspartate aminotransferase catalyzes the reaction responsible for L-glutamate transformation into L-aspartate, while 4-hydroxy-tetrahydrodipicolinate synthase is a crucial rate-limiting enzyme in the L-lysine synthesis pathway (Li et al., 2021). Our integrated analysis revealed that expression of aspartate aminotransferase (*aspB*) and aspartate-semialdehyde dehydrogenase (*asd*) were, respectively, upregulated 2.19- and 2.71-fold at the transcription level, while 4-hydroxy-tetrahydrodipicolinate synthase (*dapA*) and amidohydrolase (*dapE*) were, respectively, upregulated 1.64- and 1.34-fold at the protein level (Table 2). The upregulation of these enzymes could redirect metabolic intermediates into the L-lysine biosynthesis pathway. However, diaminopimelate epimerase (*dapE*), which catalyzes meso-2,6-diaminopimelate synthesis from LL-2,6-diaminopimelate, was downregulated 1.4-fold in WG-608, although its transcription was not obviously different from that in M-Z18.

It is noteworthy that the upregulation of several other genes could also have positive effects on L-lysine production. For example, saccharopine dehydrogenase (*LYS1*), which catalyzes L-lysine formation from saccharopine, was significantly upregulated at the mRNA level by 11.5-fold in WG-608 (Table 2), while adenylosuccinate synthase (*purA*) and adenylosuccinate lyase (*purB*) transcription was also increased by 3.11- and 2.99-fold. These alterations could lead to accelerated conversion of fumarate into L-aspartate and redirection of carbon from the TCA cycle into L-lysine biosynthesis (Figure 3). Ultimately, the L-lysine supply in WG-608 was significantly increased over that in M-Z18.

#### 3.4.8. Biosynthesis of other amino acids

In addition to enhanced expression of L-lysine related genes, we could not exclude the possibility of decreased expression in competing pathways that benefitted ε-PL production. Thus, we next investigated the expression of DEPs and DEGs in pathways that compete with L-lysine for carbon and energy. We found that enzymes that participate in synthesis of aspartate-family amino acids, such as asparagine synthase (*asnB*) in the asparagine branch, 5-methyltetrahydrofolate-homocysteine methyltransferase (*metH*) in the L-methionine branch, as well as homoserine kinase type II (*thrB2*) and threonine synthase (*thrC*) in the L-threonine branch, were all transcriptionally downregulated (Supplementary Table S5). It is thus possible that suppression of these pathways might result in concentrating carbon flux through L-aspartate toward L-lysine production.

Due to its role as the amino donor in L-lysine synthesis, L-glutamate is essential component of ε-PL biosynthesis. Our results showed downregulation of the 2-oxoglutarate dehydrogenase complex in WG-608 at the protein level, suggesting redirection of carbon flux towards L-glutamate biosynthesis (Supplementary Table S5), which is consistent with our observations of increased intracellular L-glutamate concentration (Figure 1C). Surprisingly, L-glutamate biosynthesis appears limited in WG-608 due to suppression glutamate dehydrogenase (*gdhA*) and L-glutamine synthetase (*glnA*), and further suggests that the addition of L-glutamate could further enhance ε-PL production in this strain. In addition, glutamate N-acetyltransferase (*argA*), argininosuccinate synthase (*argG*), and dapdiamide synthase (*argH*) in the L-arginine biosynthesis pathway were all transcriptionally upregulated, indicating a substantial increase in the supply of L-arginine in WG-608. The increased availability of L-arginine could lead to a cellular microenvironment with improved tolerance of acid stress conducive to ε-PL fermentation (Pan et al., 2019a,b; Wang C. Y. et al., 2020; Wang A. X. et al., 2020).

The biosynthesis of other amino acids was also changed in WG608. For instance, acetolactate synthase (*ilvB*), ketol-acid reductoisomerase (*ilvC*), 2-isopropylmalate synthase (*leuA*), 3-isopropylmalate dehydrogenase (*leuB*), and 3-isopropylmalate (*leuC*) protein and mRNA were both significantly downregulated, while aminotransferase (*ilvE*) protein expression was also reduced. This result suggested that the biosynthesis of L-isoleucine, L-leucine and L-valine was repressed, which further reduced pyruvate degradation in WG-608. Similarly, 2-oxoglutarate reductase (*serA*), phosphoserine phosphatase RsbU/P (*serB*), glycine hydroxymethyltransferase (*glyA*), and tryptophan synthase beta chain (*trpB*) were downregulated at the protein level, while *serA* and *trpB* transcription were also downregulated, suggesting the inhibition L-serine, L-glycine, and L-tryptophan biosynthesis. Additionally, the evident decline in mRNA and protein levels of transketolase (*tktA*) could limit the synthesis of Erythrose-4P, and consequently, the formation of chorismate, a precursor of L-phenylalanine and L-tryptophan. Histidinol-phosphate aminotransferase (*hisC*), which functions in the biosynthetic pathways of both L-histidine and L-phenylalanine, was downregulated by 1.23-fold at the protein level. Overall, transcriptional and translational regulation of genes involved in the biosynthesis of amino acids other than lysine contribute to WG-608 function as a highly efficient microbial cell factory for L-lysine and ε-PL production.

#### 3.4.9. ε-PL biosynthesis

ε-PL is synthesized *via* polymerization of L-lysine by a membrane-bound non-ribosomal peptide synthase (NRPS)-like ε-PL synthetase (Yamanaka et al., 2008). As previously reported, the first step in the L-lysine polymerization reaction is activation of L-lysine to produce lysyl-O-AMP at the adenylation domain of ε-PL synthetase. Here, a lysine-tRNA ligase (KARS1) that can also catalyze L-lysine monomer to form lysyl-O-AMP, was found to be upregulated by 2.47- and 1.93-fold, at the mRNA and protein levels, respectively. Upregulation of this enzyme is likely to provide a greater pool of the activated substrate lysyl-O-AMP for high-efficiency biosynthesis of ε-PL. In addition, ε-PL synthetase in WG608 was significantly upregulated by 5.53- and 2.28-fold at the transcription and protein levels, respectively, which could directly lead to enhanced ε-PL production.

#### 3.4.10. Biosynthesis of other secondary metabolites

*Streptomyces* species can synthesize 80% of the currently used antibiotics because it possesses multiple copies of polyketide synthase (PKS) and NRPS clusters. However, few studies have investigated the diversity of antibiotics synthesized by the ε-PL-producing *S. albulus*. KEGG enrichment analysis showed that all of the most significantly altered pathways were associated with secondary metabolite synthesis. Specifically, 19 DEGs and 23 DEPs, annotated as 10 enzymes, are known to be involved in the synthesis of secondary metabolites, including ε-PL synthase (*pls*), 1 NRPS DhbF (*dhbF*), 4 polyene macrolide PKSs (*amphB*, *amphC*, *amphI*, *amphK*), 1 polyene glycosyltransferase (*amphDI*), 2 cytochrome P450 monooxygenases (*amphN*, *amphL*), 1 pimaricinolide synthase PimS1 (*pimS1*). With the exception of ε-PL synthase, all of these genes/proteins were significantly downregulated at both the mRNA and protein levels (Table 3). The downregulated enzymes were annotated as involved in amphotericin B, nystatin A1, pimaricin, candicidin D, pimaricinolide, and bacillibactin synthesis, suggesting that large pools of precursors and energy used for synthesis of byproducts were accessible for ε-PL biosynthesis, thus further contributing to the enhanced production of ε-PL by WG608. Recent studies have shown that tetramycin and tetrin may function together as co-producers ε-PL in *S. albulus*, while targeted inactivation of the tetramycin and tetrin gene clusters led to a 20% increase in ε-PL (Yamanaka et al., 2019). Therefore, weakening the biosynthesis pathways of these products may provide some candidate knockdown targets for the promotion of ε-PL biosynthesis.

**Table 3:** Differential expression genes and proteins related to secondary metabolism.

Gene name	Gene ID	Entry	Definition	log_2_ fold change (gene)	Fold change (protein)
*cepK*	M-Z18AGL001343	K16432	Epsilon-poly-L-lysine synthase	2.46	2.28
*dhbF*	M-Z18AGL008387	K04780	Nonribosomal peptide synthetase DhbF	−11.19	−2.56
*dhbF*	M-Z18AGL007773	K04780	Nonribosomal peptide synthetase DhbF		−2.38
*dhbF*	M-Z18AGL000027	K04780	Nonribosomal peptide synthetase DhbF		−3.03
*dhbF*	M-Z18AGL007530	K04780	Nonribosomal peptide synthetase DhbF		1.28
*dhbF*	M-Z18AGL008387	K04780	Nonribosomal peptide synthetase DhbF		−1.25
*dhbF*	M-Z18AGL008379	K04780	Nonribosomal peptide synthetase DhbF		−2.08
*dhbF*	M-Z18AGL001743	K04780	Nonribosomal peptide synthetase DhbF		1.48
*dhbF*	M-Z18AGL000023	K04780	Nonribosomal peptide synthetase DhbF		1.31
*dhbF*	M-Z18AGL000022	K04780	Nonribosomal peptide synthetase DhbF		−1.52
*dhbF*	M-Z18AGL008387	K04780	Nonribosomal peptide synthetase DhbF		−10
*amphB*	M-Z18AGL000415	K16383	Polyene macrolide polyketide synthase, A-Type KR domains	−12.23	
*amphB*	M-Z18AGL000416	K16383	Polyene macrolide polyketide synthase, A-Type KR domains	−12.42	−2.17
*amphC*	M-Z18AGL000413	K16384	Polyene macrolide polyketide synthase	−11.56	
*amphC*	M-Z18AGL000414	K16384	Polyene macrolide polyketide synthase	−12.64	−2.5
*amphC*	M-Z18AGL000417	K16384	Polyene macrolide polyketide synthase	−13.10	
*amphI*	M-Z18AGL000425	K16385	Polyene macrolide polyketide synthase, KS-AT-KR-ACP domains	−11.58	−1.89
*amphI*	M-Z18AGL000426	K16385	Polyene macrolide polyketide synthase, KS-AT-KR-ACP domains	−12.29	
*amphI*	tr|A0A2R4PHD1|A0A2R4PHD1_STRA9	K16385	Polyene macrolide polyketide synthase, KS-AT-KR-ACP domains		−10
*amphI*	WP_079164325.1	K16385	Polyene macrolide polyketide synthase, KS-AT-KR-ACP domains		−1.59
*amphK*	M-Z18AGL000423	K16387	Polyene macrolide polyketide synthase	−12.18	
*amphK*	M-Z18AGL000424	K16387	Polyene macrolide polyketide synthase	−11.72	−2.08
*amphDI*	M-Z18AGL000418	K16388	Polyene glycosyltransferase	−12.79	−2.78
*amphDI*	M-Z18AGL007300	K16388	Polyene glycosyltransferase	−6.56	−2.5
*amphN*	M-Z18AGL000420	K16389	Cytochrome p450 monooxygenase	−9.14	−1.82
*amphN*	M-Z18AGL007298	K16389	Cytochrome p450 monooxygenase	−5.12	−2.78
*amphL*	M-Z18AGL000422	K16390	Cytochrome p450 monooxygenase	−11.78	
*amphL*	M-Z18AGL007291	K16390	Cytochrome p450 monooxygenase	−4.69	−1.54
*pimS1*	M-Z18AGL007293	K19203	Pimaricinolide synthase pims1	−5.03	
*pimS1*	M-Z18AGL007294	K19203	Pimaricinolide synthase pims1	−5.44	−2.78

#### 3.4.11. Fatty acids biosynthesis

As fatty acids are used to produce phospholipids and sophorolipids, they also represent an important class of signal molecules that participate in regulating cellular physiology and metabolic processes (Janssen and Steinbuchel, 2014). RNA-Seq and protein analysis indicated that a total of six enzymes involved in fatty acid biosynthesis were downregulated to differing degrees. In particular, malonyl-CoA:ACP transacylase (*fabD*), a critical but rate-limiting enzyme in fatty acid biosynthesis that catalyzes malonyl-ACP formation and promotes fatty acid neogenesis (Janssen and Steinbuchel, 2014), was significantly downregulated, suggesting the repression of metabolic flux from acetyl-CoA towards malonyl-CoA. Similarly, β-ketoacyl-ACP synthase III (*fabH*), which catalyzes the initial fatty acid elongation step, was strongly downregulated by 2.91-fold at the mRNA level and 1.43-fold at the protein level, resulting in decreased availability of β-ketoacyl-ACP (Handke et al., 2011). Moreover, the transcription and protein expression of three enzymes involved in fatty acid chain elongation, including β-ketoacyl-ACP reductase (*fabG*), β-hydroxyacyl-ACP dehydratase (*fabZ*), and enoyl-ACP reductase I (*fabI*), were significantly suppressed (Table 4), indicating that fatty acid biosynthesis in WG608 is apparently weaker than that in M-Z18.

**Table 4:** Differential expression genes and proteins related to fatty acid biosynthesis between WG-608 and M-Z18.

Gene name	Gene ID	Entry	Definition	log_2_ fold change (gene)	Fold change (protein)
*fabD*	M-Z18AGL005750	K00645	[Acyl-carrier-protein] S-malonyltransferase		−1.52
*fabF*	M-Z18AGL001224	K09458	3-Oxoacyl-[acyl-carrier-protein] synthase II	2.79	1.99
*fabF*	M-Z18AGL003920	K09458	3-Oxoacyl-[acyl-carrier-protein] synthase II	5.88	4.45
*fabF*	M-Z18AGL003922	K09458	3-Oxoacyl-[acyl-carrier-protein] synthase II	7.08	2.55
*fabF*	M-Z18AGL005747	K09458	3-Oxoacyl-[acyl-carrier-protein] synthase II	−1.03	−1.64
*fabF*	tr|X0MII1|X0MII1_STRA9	K09458	3-Oxoacyl-[acyl-carrier-protein] synthase II	5.7	1.75
*fabF*	tr|X0P2D8|X0P2D8_STRA9	K09458	3-Oxoacyl-[acyl-carrier-protein] synthase II	−15	−2.27
*fabG*	M-Z18AGL000564	K00059	3-Oxoacyl-[acyl-carrier protein] reductase	−10.97	−3.45
*fabG*	M-Z18AGL000893	K00059	3-Oxoacyl-[acyl-carrier protein] reductase	−10.12	−1.92
*fabG*	M-Z18AGL002188	K00059	3-Oxoacyl-[acyl-carrier protein] reductase		−1.23
*fabG*	M-Z18AGL002217	K00059	3-Oxoacyl-[acyl-carrier protein] reductase		−1.45
*fabG*	M-Z18AGL006328	K00059	3-Oxoacyl-[acyl-carrier protein] reductase	−1.41	
*fabG*	M-Z18AGL006845	K00059	3-Oxoacyl-[acyl-carrier protein] reductase	−2.17	−1.89
*fabG*	M-Z18AGL007130	K00059	3-Oxoacyl-[acyl-carrier protein] reductase		−1.52
*fabG*	M-Z18AGL008246	K00059	3-Oxoacyl-[acyl-carrier protein] reductase	−9.56	−1.64
*fabG*	M-Z18AGL008348	K00059	3-Oxoacyl-[acyl-carrier protein] reductase	−12.89	−2.44
*fabG*	M-Z18AGL008577	K00059	3-Oxoacyl-[acyl-carrier protein] reductase	−8.37	−2.86
*fabG*	M-Z18AGL008671	K00059	3-Oxoacyl-[acyl-carrier protein] reductase	−12.15	−2.13
*fabG*	SG-86AGL002882	K00059	3-Oxoacyl-[acyl-carrier protein] reductase		−1.22
*fabG*	WP_038516773.1	K00059	3-Oxoacyl-[acyl-carrier protein] reductase	−9.88	−2.33
*fabG*	WP_038518667.1	K00059	3-Oxoacyl-[acyl-carrier protein] reductase		−1.45
*fabG*	WP_038519330.1	K00059	3-Oxoacyl-[acyl-carrier protein] reductase	1.77	
*fabG*	WP_038520654.1	K00059	3-Oxoacyl-[acyl-carrier protein] reductase		−1.37
*fabG*	WP_038521679.1	K00059	3-Oxoacyl-[acyl-carrier protein] reductase		−1.28
*fabG*	WP_038525278.1	K00059	3-Oxoacyl-[acyl-carrier protein] reductase	−8.65	−1.72
*fabG*	WP_051661850.1	K00059	3-Oxoacyl-[acyl-carrier protein] reductase	2.91	−1.2
*fabG*	tr|A0A1A9QGV3|A0A1A9QGV3_STRA9	K00059	3-Oxoacyl-[acyl-carrier protein] reductase		2.12
*fabG*	tr|A0A1A9QKT8|A0A1A9QKT8_STRA9	K00059	3-Oxoacyl-[acyl-carrier protein] reductase	−1.41	−1.54
*fabG*	tr|A0A401QRM8|A0A401QRM8_STRA9	K00059	3-Oxoacyl-[acyl-carrier protein] reductase	−8.1	
*fabG*	tr|X0MY33|X0MY33_STRA9	K00059	3-Oxoacyl-[acyl-carrier protein] reductase	−5.52	
*fabG*	tr|X0N1Z2|X0N1Z2_STRA9	K00059	3-Oxoacyl-[acyl-carrier protein] reductase	−8.56	−2.56
*fabZ*	M-Z18AGL000795	K02372	3-Hydroxyacyl-[acyl-carrier-protein] dehydratase	−11.44	−2.44
*fabI*	M-Z18AGL006329	K00208	Enoyl-[acyl-carrier protein] reductase I	−1.14	−1.56
*fabV*	tr|A0A401R6B5|A0A401R6B5_STRA9	K00209	Enoyl-[acyl-carrier protein] reductase/trans-2-enoyl-CoA reductase (NAD+)		−1.28
*fabH*	M-Z18AGL003131	K00648	3-Oxoacyl-[acyl-carrier-protein] synthase III	−1.54	−1.43

It is also noteworthy that malonyl-ACP synthesis from acetyl-CoA requires ATP, and the reactions catalyzed by β-ketoacyl-ACP reductase and enoyl-ACP reductase I consume numerous NADPH and NADH. Considering this requirement, the weakening of fatty acid biosynthesis can increase the pools of available acetyl-CoA, NADPH, and ATP that can be redirected into L-lysine and ε-PL production. The conserved intracellular ATP can also be used for DNA damage repair and transport of macromolecules, or to fuel environmental adaptation mechanisms (Liu et al., 2020). Consequently, we hypothesized that the downregulation of fatty acid biosynthesis pathway may be another reason for the high production capacity of WG608.

#### 3.4.12. Verification of differential transcripts by qRT-PCR

**Figure 4:**
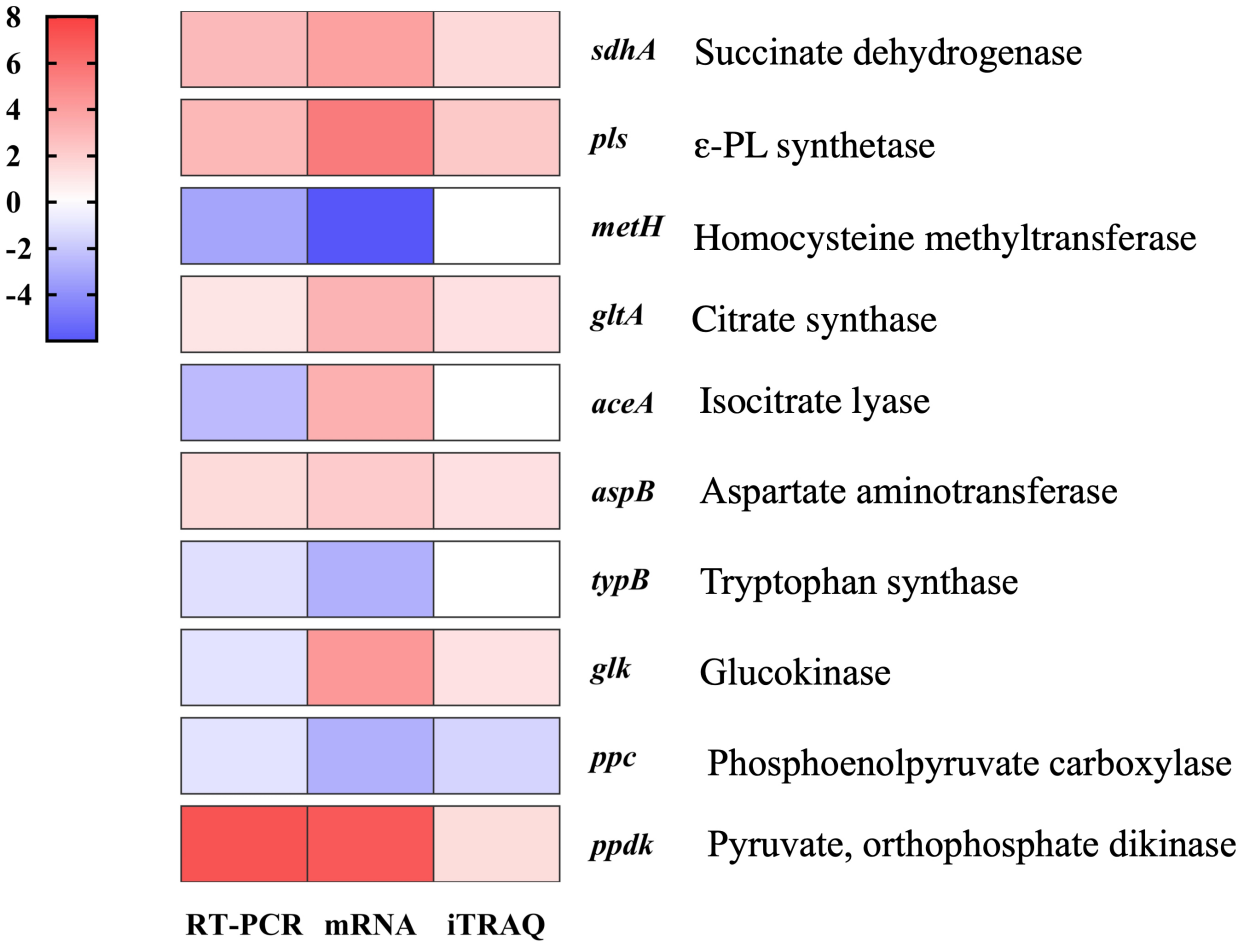
Quantitative RT-PCR verification of RNA-sequencing and iTRAQ data obtained from fed-batch fermentations of *S. albulus* WG-608 and *S. albulus* M-Z18.

To validate the reliability of our transcriptomic and proteomics data, we used qRT-PCR-based assays to evaluate the expression of 10 DEGs involved in the ε-PL biosynthesis pathway. The results showed that the transcription levels of these genes differed only slightly from the levels determined by omics analysis (Figure 4), thus supporting the reliability of the RNA-seq and iTRAQ data.

#### 3.4.13. Enhanced ATP supply in *Streptomyces albulus* WG-608 for higher ε-PL production

ε-PL is synthesized by a membrane-bounded non-ribosomal peptide synthase-like ε-PL synthetase which catalyzes the polymerization of L-lysine monomers into ε-PL. The synthesis of 1 molecule of ε-PL consumes 24–34 molecules of ATP. Although metabolic pathways connected to the ATP biosynthesis (e.g., glycolysis pathway and oxidative phosphorylation pathway) were significantly upregulated, the intercellular ATP concentrations in WG608 were still significantly lower than those in M-Z18 (Figure 1D). Hence, we speculate that ATP deficiency due to the synthesis of ε-PL in large amounts may be a major factor affecting the ε-PL production by WG-608.

**Figure 5:**
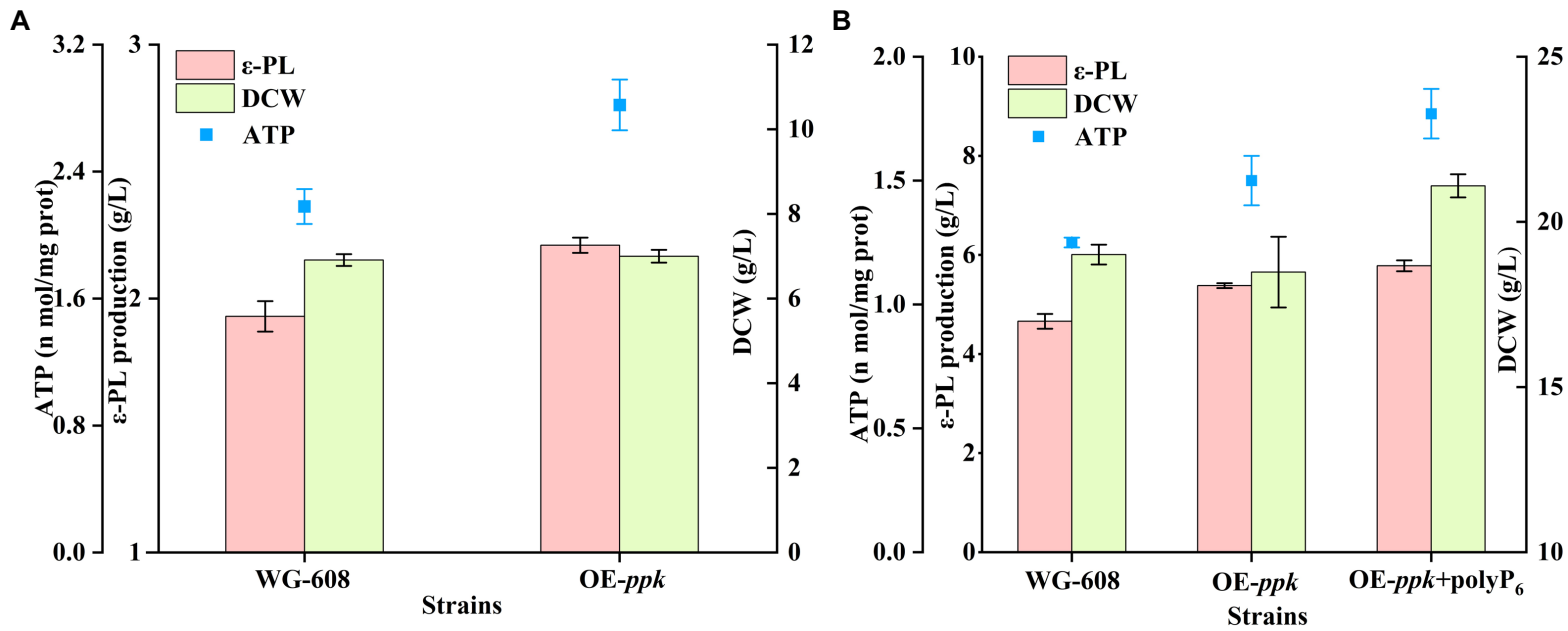
Enhancement of ε-PL production with overexpressing *ppk* gene and polyP_6_ feeding. **(A)** Shake-flask fermentation of *S. albulus* WG-608 and OE-*ppk*. **(B)** Batch-fermentation of OE-*ppk* with polyP_6_ addition and *S. albulus* WG-608. DCW, dry cell weight, red box represents ε-PL production, green box represents DCW (dry cell weight), whereas blue dot represents ATP concentration.

To verify this deduction, gene *ppk* (encoding polyphosphate kinase) was overexpressed in WG608 to enhance the ATP supply. As shown in Figures 5A,B, the intracellular ATP concentration in OE-*ppk* was increased by 28.90% compared with that in WG-608. The ε-PL production of OE-*ppk* reached 2.21 ± 0.04 and 5.38 g/L in shake flask and 1-L bioreactor, which was 14.51 and 15.45% higher than that in WG-608. Since polyphosphate kinase catalyzes the biosynthesis of ATP with ADP and polyP as substrates, 1 g/L polyP_6_ was added in the 1-L fermentation for further increasing the intracellular ATP concentration of OE-*ppk*. As a result, ε-PL production was further improved by 24.03% compared to that of WG-608 (Figure 5B). These suggest that the inadequate ATP supply is an important limiting factor in ε-PL biosynthesis by WG-608.

## 4. Conclusion

We comparatively analyzed the physiological, transcriptomic and proteomic changes and proposed the responsible a molecular mechanism for elevated ε-PL production in *S. albulus* WG-608. As shown in Figure 3, the upregulation of glycolysis pathway, pentose phosphate pathway provides sufficient NADPH and oxaloacetate to accommodate increased L-lysine biosynthesis, while the upregulated oxidative phosphorylation increases the supply of ATP necessary for ε-PL biosynthesis from L-lysine. The upregulated MtrAB promotes DNA synthesis and subsequently increases transcription and translation processes that enable higher ε-PL biosynthesis by *S. albulus* WG-608. The upregulation of MprAB and PepD positively regulate *sigE* and *sigB*, which could further activate *pls* transcription and ε-PL biosynthesis. Biosynthetic pathways for fatty acids, L-histidine, L-tryptophan, L-valine, and L-leucine, and some secondary metabolite by-products are all downregulated at the mRNA and protein levels, which conserves and redirects energy and precursors for ε-PL production. Meanwhile, *S. albulus* WG-608 tolerance of adverse extracellular conditions is also enhanced. The upregulation of VanJ and VanS enhanced the drug resistance of WG-608. The upregulated glutamate decarboxylase system and increased concentrations of L-arginine, L-glutamate, L-aspartate, and L-lysine improved the acid resistance in WG-608. These results contribute to explaining the continuous ε-PL biosynthesis by WG-608 at low pH and high ε-PL concentrations. The physiological, transcriptomic and proteomic association analysis also implied that the high-yielding WG-608 lack of sufficient ATP for ε-PL biosynthesis. Overexpression of *ppk* improved ε-PL production in WG-608, and the addition of polyP_6_ further enhanced the intracellular ATP supply and ε-PL production. These cumulative findings provide a clear picture of the molecular basis for high ε-PL biosynthesis, while also laying a theoretical foundation for the development of advanced microbial cell factories for industrial-scale production of ε-PL. To our knowledge, this is the first report on enhancing ε-PL production *via* improving ATP supply.

## Data availability statement

The data presented in the study are deposited in the National Center for Biotechnology Information repository can be found at: https://submit.ncbi.nlm.nih.gov/subs/bioproject/SUB11455437/overview, BioProject ID: PRJNA869557. The mass spectrometry proteomics data have been deposited to the ProteomeXchange Consortium via the PRIDE partner repository with the dataset identifier PXD036099.

## Author contributions

LW designed all the experiments and drafted this manuscript. LW and MW performed physiological and fermentation profiles. LW and HY conducted multi-omics sequencing. JZ, HZ, ZM, and XC revised this manuscript. All authors contributed to the article and approved the submitted version.

## Funding

This work was supported by the following: National Key R&D Program of China (2020YFA0907700); National Natural Science Foundation of China (31901622 and 31671846); Natural Science Foundation of Jiangsu Province (BK20190585 and BK20191332); The Fundamental Research Funds for the Central Universities (JUSRP123040); Program of the Key Laboratory of Industrial Biotechnology, Ministry of Education, China (KLIB-KF202206 and KLIB-KF202204); Program of Introducing Talents of Discipline to Universities (111-2-06).

## Conflict of interest

The authors declare that the research was conducted in the absence of any commercial or financial relationships that could be construed as a potential conflict of interest.

## Publisher’s note

All claims expressed in this article are solely those of the authors and do not necessarily represent those of their affiliated organizations, or those of the publisher, the editors and the reviewers. Any product that may be evaluated in this article, or claim that may be made by its manufacturer, is not guaranteed or endorsed by the publisher.

## Footnotes

^1^http://geneontology.org/

^2^www.kegg.jp/kegg/kegg1.html
