# Supplementary material for: Integrative transcriptome and proteome revealed high-yielding mechanisms of epsilon-poly-L-lysine by Streptomyces albulus
Source: Front Microbiol. 2023 Apr 20;14:1123050. doi: 10.3389/fmicb.2023.1123050 (PMC10157215; doi:10.3389/fmicb.2023.1123050)
Supplement: Supplementary file 1 [file Data_Sheet_1.docx]

**Integrative transcriptome and proteome revealed high-yielding mechanisms of epsilon-poly-L-lysine by *Streptomyces albulus***

Liang Wang^1^, Hao Yang^1^, Mengping Wu^1^, Jianhua Zhang^1^, Hongjian Zhang^1^, Zhonggui Mao^1^, Xusheng Chen^1^^[[1]](#footnote-1)^*

^1^The Key Laboratory of Industrial Biotechnology, Ministry of Education, School of Biotechnology, Jiangnan University, 1800 Lihu Avenue, Wuxi 214122, Jiangsu, China

*** Correspondence:**

Xusheng Chen

[chenxs@jiangnan.edu.cn](mailto:chenxs@jiangnan.edu.cn)


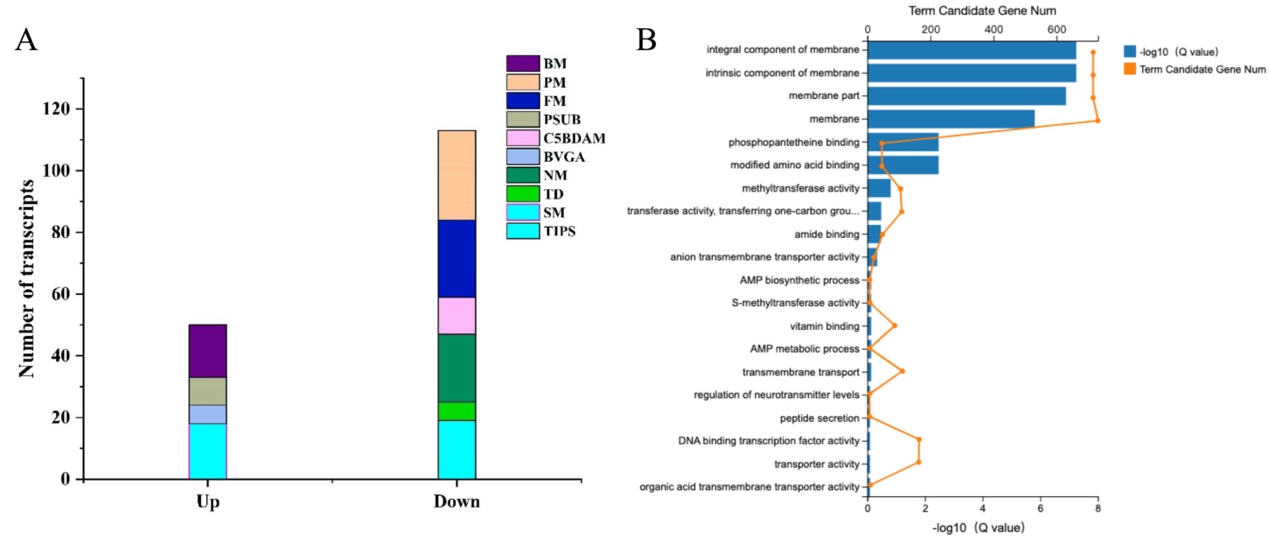


**FIGURE S1 Differentially expressed genes (DEGs) between ε-PL high-producing mutant *S. albulus* WG-608 and the original strain *S. albulus* M-Z18.** (A) The top 10 significantly enriched KEGG pathways. BM, Biotin metabolism; PM, Phenylalanine metabolism; FM, Fatty acid biosynthesis; PSUB, Polyketide sugar unit biosynthesis; C5BDAM, C5-Branched dibasic acid metabolism; BVGA, Biosynthesis of vancomycin group antibiotics; NM, Nitrogen metabolism; TD, Toluene degradation; SM, Sulfur metabolism; TIPS, Type I polyketide structures. (B) The top 20 functional characterization of the DEGs. Y-axis demonstrates functional categories of DEGs, up x-axis demonstrates the number of DEGs, and the down x-axis demonstrates the level of -Log (P value).


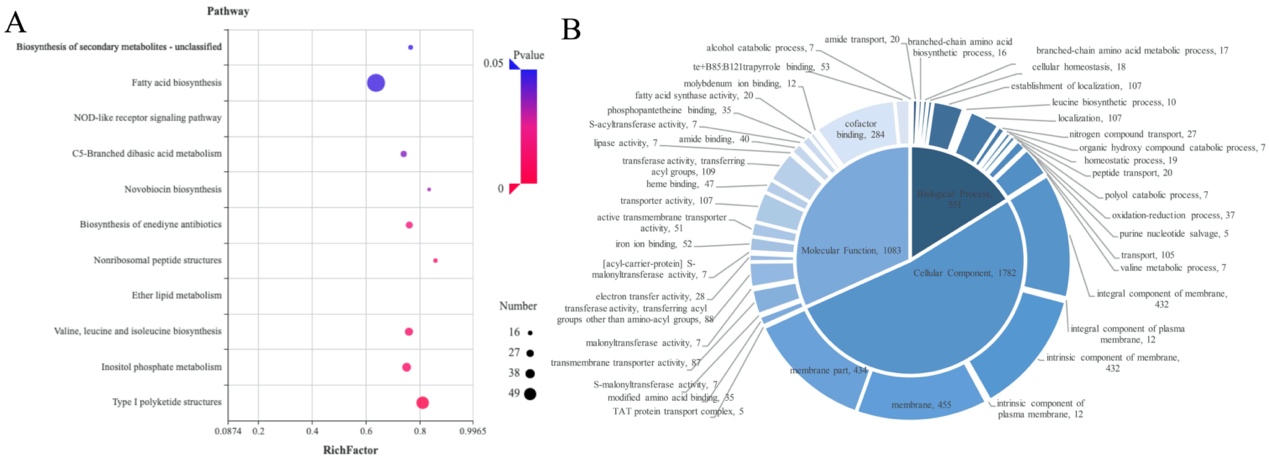


**FIGURE S2 Differentially expressed proteins (DEPs) between mutant *S. albulus* WG-608 and the original strain *S. albulus* M-Z18.** (A) The top 10 significantly enriched KEGG pathways. (B) GO Enrichment Analysis assigned to the DEPs. All DEPs are classified into biological process, cellular component and molecular function between *S. albulus* WG-608 and *S. albulus* M-Z18. Number of DEPs related to GO terms follows the name of each term.

**Table S1** Primers used in this study.

| **Primers** | **Sequence (5’-3’)** | **Description** |
| --- | --- | --- |
| P-F | ATGCGTCCGGCGTAGAGGAT | Validation primers |
| P-R | CTCGATGGCGTCGTTGATGGT |  |

**TABLE S2** Lists of primers for qRT-PCR validation.

| Genes | F-primer | R-primer |
| --- | --- | --- |
| *aceA* | CTCACCGTCCTTCAACTG | CGTGATGAACTGGAACTTG |
| *sdhA*  *pls* | AAGTACGGCAACCTCTTCG | GTAGATCCGCATGGGCAC |
|  | CTTCACGGTGAGCGAGCA | GAGCGATTCGACGAAGGTG |
| *gltA* | ATGTTCACGTCCATGTTCAC | TCCGGTTCGCTTCTGTTC |
| *aspB* | CACGATCCGCACGACACC | CAGATCGGTCAGCACCAC |
| *metH* | GAGAAGTCCGACGATGAG | TTCTTACCGATGTCATGGA |
| *typB* | TCATCCTGTTCAACCTCA | CATGTCCGTCATGTTTCC |
| *glk* | CAGCCCAGTCAACATCCC | GAGACCACCATGCACAGG |
| *ppc* | GCAACTTCCTCTCCAACG | CTTGATGACGGCGAAGAC |
| *ppdk* | GACACCGTCCTCAACATC | CTTGCCGAACATCTGGAT |

**TABLE S3** Differential expression proteins related to signal transduction.

|  | Gene Name | Gene ID | Entry | Definition | Fold change (protein) |
| --- | --- | --- | --- | --- | --- |
| Two-component system | *mtrA* | M-Z18AGL003955 | K07670 | Two-component system, OmpR family, response regulator MtrA | 1.28 |
|  | *mtrA* | M-Z18AGL001293 | K07670 | Two-component system, OmpR family, response regulator MtrA | 1.82 |
|  | *mtrA* | M-Z18AGL001678 | K07670 | Two-component system, OmpR family, response regulator MtrA | 1.91 |
|  | *mtrA* | M-Z18AGL005226 | K02483 | Two-component system, OmpR family, response regulator MtrA | -1.96 |
|  | *mtrB* | WP_038523719.1 | K07654 | Two-component system, OmpR family, sensor histidine kinase MtrB | 1.53 |
|  | *mtrB* | M-Z18AGL005352 | K07654 | Two-component system, OmpR family, sensor histidine kinase MtrB | 1.52 |
|  | *mtrB* | M-Z18AGL001679 | K07654 | Two-component system, OmpR family, sensor histidine kinase MtrB | 1.86 |
|  | *mtrB* | M-Z18AGL003438 | K07654 | Two-component system, OmpR family, sensor histidine kinase MtrB | 1.31 |
|  | *mtrB* | tr\|A0A1A9QUJ3\|A0A1A9QUJ3_STRA9 | K07654 | Two-component system, OmpR family, sensor histidine kinase MtrB | 2.16 |
|  | *mtrB* | M-Z18AGL001046 | K07654 | Two-component system, OmpR family, sensor histidine kinase MtrB | 1.27 |
|  | *mtrB* | M-Z18AGL003956 | K07654 | Two-component system, OmpR family, sensor histidine kinase MtrB | 1.23 |
|  | *qseC* | SG-86AGL000799 | K07645 | Two-component system, OmpR family, sensor histidine kinase MtrB | 2.77 |
|  | *phoP* | M-Z18AGL005417 | K07658 | Two-component system, OmpR family, alkaline phosphatase synthesis response regulator PhoP | 1.3 |
|  | *mprA* | M-Z18AGL003840 | K07669 | Two-component system, OmpR family, response regulator MprA | 1.22 |
|  | *pepD* | WP_038520942.1 | K08372 | Putative serine protease PepD | 1.55 |
|  | *PleC* | tr\|X0N3L9\|X0N3L9_STRA9 | K07716 | Two-component system, cell cycle sensor histidine kinase PleC | 2.19 |
|  | *pleC* | SG-86AGL000615 | K07716 | Two-component system, cell cycle sensor histidine kinase PleC | 3.19 |
|  | *pleC* | M-Z18AGL001050 | K07716 | Two-component system, cell cycle sensor histidine kinase PleC | -2.5 |
|  | *senX3* | M-Z18AGL003804 | K07768 | Two-component system, OmpR family, sensor histidine kinase SenX3 | 1.41 |
|  | *regX3* | M-Z18AGL005162 | K07776 | Two-component system, OmpR family, response regulator RegX3 | 1.26 |
|  | *regX4* | tr\|A0A059WBS4\|A0A059WBS4_STRA9 | K07776 | Two-component system, OmpR family, response regulator RegX3 | 1.48 |
|  | *qseC* | SG-86AGL000799 | K07645 | Two-component system, OmpR family, sensor histidine kinase QseC | 2.77 |
|  | pheS | M-Z18AGL006612 | K01889 | Two-component system, OmpR Family, phosphate regulon sensor histidine kinase | 1.21 |
|  | *devR* | M-Z18AGL008540 | K07695 | Two-component system, NarLfamily, response regulator DevR | -3.3 |
|  | *devR* | WP_038516964.1 | K07695 | Two-component system, NarLfamily, response regulator DevR | -2.04 |
|  | *degU* | SG-86AGL000347 | K07692 | Two-component system, NarL family, response regulator DegU | 1.35 |
|  | *comA* | M-Z18AGL007288 | K07691 | two-component system, NarL family, competent response regulator ComA | -3.57 |
|  | *devS* | WP_037634682.1 | K07682 | Two-component system, NarL family, sensor histidine kinase DevS | -3.45 |
|  | *pstS* | M-Z18AGL003884 | K02040 | Phosphate transport system substrate-binding protein | 2.14 |
|  | *phoD* | tr\|A0A1A9QQ54\|A0A1A9QQ54_STRA9 | K01113 | Alkaline Phosphatase D | 1.5 |
|  | *phoD* | SG-86AGL005843 | K01113 | Alkaline Phosphatase D | -1.23 |
|  | *phoD* | WP_107080247.1 | K01113 | Alkaline Phosphatase D | 2.17 |
|  | *fixL* | tr\|A0A059WBW8\|A0A059WBW8_STRA9 | K14986 | Two-component system, LuxR family, sensor kinase FixL | 1.57 |
|  | *fadR* | M-Z18AGL007986 | K13770 | TetR/AcrR family transcriptional regulator, fatty acid metabolism regulator protein | -2 |
|  | *desK* | M-Z18AGL001751 | K07778 | Two-component system, NarL family, sensor histidine kinase DesK | 1.5 |
|  | *desK* | M-Z18AGL005950 | K07778 | Two-component system, NarL family, sensor histidine kinase DesK | 1.32 |
|  | *desK* | M-Z18AGL003574 | K07778 | Two-component system, NarL family, sensor histidine kinase DesK | 1.82 |
|  | *desK* | WP_038523697.1 | K07778 | Two-component system, NarL family, sensor histidine kinase DesK | 1.22 |
|  | *desK* | SG-86AGL004124 | K07778 | Two-component system, NarL family, sensor histidine kinase DesK | 1.26 |
|  | *desK* | WP_051661609.1 | K07778 | Two-component system, NarL family, sensor histidine kinase DesK | -1.35 |
|  | *desK* | M-Z18AGL007120 | K19694 | Two-component system, sensor histidine kinase ChiS | 1.86 |
|  | *degS* | M-Z18AGL003984 | K07777 | Two-component system, NarL family, sensor histidine kinase DegS | 1.3 |
|  | *degS* | WP_106970503.1 | K07777 | Two-component system, NarL family, sensor histidine kinase DegS | -1.56 |
|  | *aphA* | M-Z18AGL000460 | K10917 | PadR family transcriptional regulator, regulatory protein AphA | -1.96 |
|  | *fixL* | tr\|A0A059WBW8\|A0A059WBW8_STRA9 | K14986 | Two-component system, LuxR family, sensor kinase FixL | 1.57 |
|  | *dnr* | tr\|A0A1A9QUZ0\|A0A1A9QUZ0_STRA9 | K21563 | CRP/FNR family transcriptional regulator, dissimilatory nitrate respiration regulator | 3.38 |
|  | *vanJ* | M-Z18AGL005902 | K18353 | Vancomycin resistance protein VanJ | 2.74 |
|  | *vanRAc* | tr\|X0N1T4\|X0N1T4_STRA9 | K18352 | Two-component system, OmpR family, response regulator VanR | -1.23 |
|  | *vanSAc* | M-Z18AGL000538 | K18351 | Two-component system, OmpR family, sensor histidine kinase VanS | 2.06 |
| Quorum sensing | *degU* | SG-86AGL000347 | K07692 | NarL family, response regulator DegU | 1.35 |
|  | *degU* | M-Z18AGL004894 | K07692 | NarL family, response regulator DegU | 1.83 |
|  | *AphA* | M-Z18AGL000460 | K10917 | PadR family transcriptional regulator, regulatory protein AphA | -1.96 |
|  | *AphA* | M-Z18AGL000957 | K10917 | PadR family transcriptional regulator, regulatory protein AphA | -2.7 |
|  | *AphA* | M-Z18AGL000545 | K10917 | PadR family transcriptional regulator, regulatory protein AphA | -1.41 |
|  | *fhrR* | M-Z18AGL003417 | K20325 | TetR/AcrR family transcriptional regulator, Clp-modulated transcription factor | -1.45 |
|  | *fhrR* | tr\|A0A059WFF1\|A0A059WFF1_STRA9 | K20325 | TetR/AcrR family transcriptional regulator, Clp-modulated transcription factor | -2.38 |
|  | *fhrR* | M-Z18AGL004678 | K20325 | TetR/AcrR family transcriptional regulator, Clp-modulated transcription factor | 1.76 |
|  | *fhrR* | M-Z18AGL007934 | K20325 | TetR/AcrR family transcriptional regulator, Clp-modulated transcription factor | -3.45 |
|  | *fhrR* | WP_051661550.1 | K20325 | TetR/AcrR family transcriptional regulator, Clp-modulated transcription factor | -5 |
|  | *fhrR* | tr\|A0A401QPZ9\|A0A401QPZ9_STRA9 | K20325 | TetR/AcrR family transcriptional regulator, Clp-modulated transcription factor | -2 |
|  | *fhrR* | M-Z18AGL007986 | K13770 | TetR/AcrR family transcriptional regulator, transcriptional repressor of bet genes | -2 |
|  | *fixL* | tr\|A0A059WBW8\|A0A059WBW8_STRA9 | K14986 | LuxR family, sensor kinase FixL | 1.57 |
|  | *degS* | M-Z18AGL003984 | K07777 | NarL family, sensor histidine kinase DegS | 1.3 |
|  | *degS* | WP_106970503.1 | K07777 | NarL family, sensor histidine kinase DegS | -1.56 |
|  | *qseC* | SG-86AGL000799 | K07645 | OmpR family, sensor histidine kinase QseC | 2.77 |
|  | *pleC* | tr\|X0N3L9\|X0N3L9_STRA9 | K07716 | OmpR family, cell cycle sensor histidine kinase PleC | 2.19 |
|  | *pleC* | SG-86AGL000615 | K07716 | OmpR family, cell cycle sensor histidine kinase PleC | 3.19 |
|  | *pleC* | M-Z18AGL001050 | K07716 | OmpR family, cell cycle sensor histidine kinase PleC | -2.5 |
|  | *pknK* | M-Z18AGL000238 | K13419 | Serine/threonine-protein kinase PknK | -2.13 |
|  | *prkC* | M-Z18AGL001277 | K12132 | Eukaryotic-like serine/Threonine-protein kinase | 1.84 |
|  | *dnr* | tr\|A0A1A9QUZ0\|A0A1A9QUZ0_STRA9 | K21563 | CRP/FNR family transcriptional regulator, cyclic AMP receptor protein | 3.38 |
|  | *sph* | WP_038523331.1 | K01117 | Phospholipase C | 2.91 |
|  | *plc* | M-Z18AGL004729 | K01114 | Phospholipase C | 3.66 |
|  | *plc* | WP_038521897.1 | K01114 | Phospholipase C | 3.28 |
|  | *plc* | M-Z18AGL002349 | K01114 | Phospholipase C | 2.87 |
|  | *plc* | M-Z18AGL000864 | K01114 | Phospholipase C | -2.63 |
|  | *toxC* | M-Z18AGL000297 | K20332 | Toxoflavin biosynthesis protein ToxC | -2.63 |
|  | *toxD* | WP_045788673.1_dup | K20333 | Toxoflavin biosynthesis protein ToxD | -1.67 |
|  | *toxA* | WP_038527472.1 | K20331 | Toxoflavin synthase | -6.25 |
|  | *trpE* | M-Z18AGL006123 | K01657 | Anthranilate synthase component I | -1.37 |
|  | *gadB* | M-Z18AGL007261 | K01580 | Glutamate decarboxylase | 1.28 |
|  | *—* | SG-86AGL006742 | K22902 | D-Galactosaminyltransferase, putative drug exporter of the MD superfamily | 2.02 |
|  | *livH* | M-Z18AGL006168 | K01997 | Branched-chain amino acid transport system permease protein | 2.38 |
|  | *livK* | M-Z18AGL007419 | K01999 | Branched-chain amino acid transport system substrate-binding protein | 1.23 |
|  | *ABC.SP.S* | WP_078487435.1 | K02055 | Putative spermidine/putrescine transport system substrate-binding protein | 1.27 |
|  | *ABC.SP.A* | SG-86AGL001774 | K02052 | Putative spermidine/putrescine transport system ATP-binding protein | 1.37 |
| Protein export | *secD* | M-Z18AGL006698 | K03072 | Preprotein translocase subunit SecD | 1.66 |
|  | *secF* | M-Z18AGL006699 | K03074 | Preprotein translocase subunit SecF | 1.98 |
|  | *secG* | tr\|A0A1A9QG95\|A0A1A9QG95_STRA9 | K03075 | Preprotein translocase subunit SecG | 1.52 |
|  | *yajC* | SG-86AGL002071 | K03210 | Preprotein translocase subunit YaJc | 2.02 |
|  | *secY* | tr\|A0A059W3C8\|A0A059W3C8_STRA9 | K03076 | Preprotein translocase subunit C | 1.39 |
|  | *lepB* | SG-86AGL005926 | K03100 | Signal peptidase I | 1.98 |
|  | *lepB* | M-Z18AGL002799 | K03100 | Signal peptidase I | 1.25 |
|  | *tatA* | M-Z18AGL006579 | K03116 | Sec-independent protein translocase protein TatA | -1.45 |
|  | *tatB* | M-Z18AGL002657 | K03117 | Sec-independent protein translocase protein TatB | -2.27 |
|  | *tatB* | M-Z18AGL003250 | K03117 | Sec-independent protein translocase protein TatB | 2.09 |
|  | *tatC* | WP_038518429.1 | K03118 | Sec-independent protein translocase protein TatC | 1.41 |
|  | *yidC* | M-Z18AGL004401 | K03217 | Yidc/Oxa1 family membrane protein insertase | 1.81 |
|  | *yidC* | tr\|A0A1A9QR96\|A0A1A9QR96_STRA9 | K03217 | Yidc/Oxa2 family membrane protein insertase | 2.03 |
|  | *vgrG* | WP_038515859.1 | K11904 | Type VI secretion system secreted protein VgrG | -2.94 |
|  | *virD4* | M-Z18AGL002132 | K03205 | Type IV secretion system protein VirD4 | -1.92 |

**TABLE S4** Differential expression proteins related to ABC transporters.

| **Gene Name** | **Gene ID** | **Entry** | **Definition** | **Fold change**  **(Protein)** |
| --- | --- | --- | --- | --- |
| *ABC-2.A* | WP_037682663.1 | K01990 | ABC-2 type transport system ATP-binding protein | 1.31 |
| *ABC-2.A* | M-Z18AGL003016 | K01990 | ABC-2 type transport system ATP-binding protein | 1.21 |
| *ABC-2.A* | M-Z18AGL006101 | K01990 | ABC-2 type transport system ATP-binding protein | -1.37 |
| *ABC-2.A* | M-Z18AGL006244 | K01990 | ABC-2 type transport system ATP-binding protein | -1.41 |
| *ABC-2.A* | M-Z18AGL004319 | K01990 | ABC-2 type transport system ATP-binding protein | 1.41 |
| *ABC-2.A* | M-Z18AGL005361 | K01990 | ABC-2 type transport system ATP-binding protein | 1.97 |
| *ABC-2.A* | M-Z18AGL004627 | K01990 | ABC-2 type transport system ATP-binding protein | -2.44 |
| *ABC-2.A* | M-Z18AGL003274 | K01990 | ABC-2 type transport system ATP-binding protein | 1.44 |
| *ABC-2.P* | M-Z18AGL003386 | K01992 | ABC-2 type transport system permease protein | 2.21 |
| *ABC-2.P* | tr\|A0A1A9QRM4\|A0A1A9QRM4_STRA9 | K01992 | ABC-2 type transport system permease protein | 1.61 |
| *ABC-2.P* | M-Z18AGL004788 | K01992 | ABC-2 type transport system permease protein | 1.23 |
| *ABC-2.P* | M-Z18AGL005476 | K01992 | ABC-2 type transport system permease protein | -1.54 |
| *ABC.CD.A* | SG-86AGL001007 | K02003 | Putative ABC transport system ATP-binding protein | 2.31 |
| *ABC.CD.A* | WP_078488782.1 | K02003 | Putative ABC transport system ATP-binding protein | -1.75 |
| *ABC.CD.A* | tr\|A0A1A9QXB1\|A0A1A9QXB1_STRA9 | K02003 | Putative ABC transport system ATP-binding protein | -1.54 |
| *ABC.CD.A* | tr\|A0A401R8D8\|A0A401R8D8_STRA9 | K02003 | Putative ABC transport system ATP-binding protein | 1.49 |
| *ABC.CD.A* | M-Z18AGL001691 | K02003 | Putative ABC transport system ATP-binding protein | 2.19 |
| *ABC.CD.P* | M-Z18AGL005129 | K02004 | Putative ABC transport system permease protein | 2.01 |
| *ABC.CD.P* | tr\|A0A1A9QWD5\|A0A1A9QWD5_STRA9 | K02004 | Putative ABC transport system permease protein | -1.75 |
| *ABC.CD.P* | WP_016579193.1 | K02004 | Putative ABC transport system permease protein | 1.55 |
| *ddpF* | M-Z18AGL003285 | K02032 | Peptide/Nickel transport system ATP-binding protein | -1.27 |
| *ddpF* | M-Z18AGL000479 | K02032 | Peptide/Nickel transport system ATP-binding protein | 1.39 |
| *ddpF* | WP_038525406.1 | K02032 | Peptide/Nickel transport system ATP-binding protein | 1.33 |
| *ddpF* | M-Z18AGL003284 | K02032 | Peptide/Nickel transport system ATP-binding protein | -1.54 |
| *ddpF* | tr\|A0A1A9QJ93\|A0A1A9QJ93_STRA9 | K02032 | Peptide/Nickel transport system ATP-binding protein | 1.79 |
| *lolD* | M-Z18AGL001116 | K09810 | Lipoprotein-releasing system ATP-binding protein | 1.93 |
| *atpB* | M-Z18AGL003057 | K02108 | F-type H^+^-transporting ATPase subunit a | 1.27 |
| *atpA* | WP_038521770.1 | K02111 | F-type H^+^/Na^+^-transporting ATPase subunit alpha | 1.84 |
| *atpE* | M-Z18AGL003056 | K02110 | F-type H^+^-transporting ATPase subunit c | 1.48 |

**TABLE S5** Differential expression genes and proteins associated with the biosynthesis of other amino acids.

| **Gene Name** | **Gene ID** | **Entry** | **Definition** | **log_2_ Fold change (Gene)** | **Fold change (Protein)** |
| --- | --- | --- | --- | --- | --- |
| *hisC* | M-Z18AGL006283 | K00817 | Histidinol-phosphate aminotransferase |  | -1.23 |
| *tyrA1* | M-Z18AGL003639 | K04093 | Chorismate mutase | 1.63 |  |
| *tyrA1* | M-Z18AGL008388 | K04093 | Chorismate mutase | -13.03 |  |
| *phhA* | M-Z18AGL001391 | K00500 | Phenylalanine-4-hydroxylase | 2.86 |  |
| *trpE* | M-Z18AGL006123 | K01657 | Anthranilate synthase component I |  | -1.37 |
| *glyA* | M-Z18AGL003060 | K00600 | Glycine hydroxymethyltransferase |  | -1.23 |
| *glyA* | M-Z18AGL002941 | K00600 | Glycine hydroxymethyltransferase |  | -1.23 |
| *aroK* | M-Z18AGL006720 | K00891 | Shikimate kinase |  | -1.23 |
| *trpB* | M-Z18AGL006128 | K01696 | Tryptophan synthase beta chain |  | -1.56 |
| *trpB* | M-Z18AGL007520 | K06001 | Tryptophan synthase beta chain | -1.57 |  |
| *serA* | M-Z18AGL002895 | K00058 | D-3-phosphoglycerate dehydrogenase/2-oxoglutarate reductase | -1.35 | -1.75 |
| *serB* | M-Z18AGL003270 | K07315 | Phosphoserine phosphatase RsbU/P |  | -1.37 |
| *tdcB* | M-Z18AGL000555 | K01754 | Threonine dehydratase | -10.39 | -1.61 |
| *tdcB* | M-Z18AGL003387 | K01754 | Threonine dehydratase |  |  |
| *tdcB* | M-Z18AGL007099 | K01754 | Threonine dehydratase |  |  |
| *tdcG* | M-Z18AGL002942 | K01752 | L-serine dehydratase | 1.51 | 1.39 |
| *CBS* | tr\|X0P197\|X0P197_STRA9 | K01697 | Cystathionine beta-synthase |  | 1.3 |
| *ala* | M-Z18AGL007431 | K19244 | Alanine dehydrogenase | 1.62 |  |
| *alaA* | M-Z18AGL001502 | K14260 | Alanine-synthesizing transaminase |  | 1.45 |
| *ilvB* | M-Z18AGL008103 | K01652 | Acetolactate synthase I/II/III large subunit | -7.89 |  |
| *ilvB* | M-Z18AGL002892 | K01652 | Acetolactate synthase I/II/III large subunit | -1.37 |  |
| *ilvB* | M-Z18AGL002893 | K01652 | Acetolactate synthase I/II/III large subunit | -1.53 | -1.45 |
| *ilvB* | M-Z18AGL001910 | K01652 | Acetolactate synthase I/II/III large subunit | -1.84 | -2.04 |
| *ilvC* | M-Z18AGL002891 | K00053 | Ketol-acid reductoisomerase | -1.6 | -2.5 |
| *ilvD* | M-Z18AGL003941 | K01687 | Dihydroxy-acid dehydratase |  |  |
| *ilvD* | M-Z18AGL000729 | K01687 | Dihydroxy-acid dehydratase | -6.19 |  |
| *ilvE* | M-Z18AGL000832 | K00826 | Branched-chain amino acid aminotransferase | -11.8 | -1.96 |
| *ilvE* | WP_038521930.1 | K00826 | Branched-chain amino acid aminotransferase |  | -1.39 |
| *alaA* | M-Z18AGL001502 | K14260 | alanine-synthesizing transaminase |  | 1.45 |
| *leuA* | M-Z18AGL002871 | K01649 | 2-Isopropylmalate synthase |  | -1.25 |
| *leuA* | WP_038516745.1 | K01649 | 3-Isopropylmalate synthase |  | -2.13 |
| *leuC* | M-Z18AGL000747 | K01703 | 3-isopropylmalate/(R)-2-methylmalate dehydratase large subunit | -9.68 |  |
| *leuC* | M-Z18AGL002850 | K01703 | 3-isopropylmalate/(R)-2-methylmalate dehydratase large subunit | -3.84 | -2.17 |
| *leuD* | M-Z18AGL000749 | K01704 | 3-isopropylmalate/(R)-2-methylmalate dehydratase small subunit | -9.41 | -1.75 |
| *leuD* | M-Z18AGL002849 | K01704 | 3-isopropylmalate/(R)-2-methylmalate dehydratase small subunit | -3.92 | -2.27 |
| *leuB* | M-Z18AGL000746 | K00052 | 3-Isopropylmalate dehydrogenase | -9.26 | -1.49 |
| *leuB* | M-Z18AGL002880 | K00052 | 4-Isopropylmalate dehydrogenase | -2.31 | -1.69 |
| *gdhA* | M-Z18AGL002922 | K00262 | Glutamate dehydrogenase (NADP+) | -1.01 |  |
| *gdhA* | M-Z18AGL008102 | K00261 | Glutamate dehydrogenase (NAD(P)+) | -8.72 |  |
| *glnA* | M-Z18AGL005957 | K01915 | Glutamine synthetase | -2.15 | -1.89 |
| *glnA* | M-Z18AGL005962 | K01915 | Glutamine synthetase | -1.33 | -1.35 |
| *ocd* | M-Z18AGL000396 | K01750 | Ornithine cyclodeaminase | -11.62 | -3.45 |
| *ocd* | M-Z18AGL008332 | K01750 | Ornithine cyclodeaminase | -12.35 | -3.57 |
| *ocd* | tr\|A0A401QR09\|A0A401QR09_STRA9 | K01750 | Ornithine cyclodeaminase |  | -1.89 |
| *argD* | M-Z18AGL000798 | K00821 | Acetylornithine/N-succinyldiaminopimelate aminotransferase | -11.65 | -2.44 |
| *arga/argJ* | M-Z18AGL001706 | K00620 | Glutamate N-acetyltransferase/amino-acid N-acetyltransferase | 2.92 |  |
| *argF* | M-Z18AGL002527 | K00611 | Ornithine carbamoyltransferase |  | 1.11 |
| *rocF* | M-Z18AGL001705 | K01476 | Arginase | 3.72 | 1.3 |
| *rocD* | M-Z18AGL001941 | K00819 | Ornithine-oxo-acid transaminase | 1.13 |  |
| *rocD* | M-Z18AGL001715 | K00819 | Ornithine-oxo-acid transaminase | 1.62 | 2.32 |
| *argG* | M-Z18AGL002471 | K01940 | Argininosuccinate synthase |  | 1.22 |
| *argH* | M-Z18AGL001719 | K22114 | Dapdiamide synthase | 2.17 | 1.43 |
| *proC* | M-Z18AGL003932 | K00286 | Pyrroline-5-carboxylate reductase |  | -1.49 |
| *asnB* | M-Z18AGL000619 | K01953 | Asparagine synthase (glutamine-hydrolysing) | -14.09 | -4 |
| *asnB* | M-Z18AGL007674 | K01953 | Asparagine synthase (glutamine-hydrolysing) | -3.51 | -1.96 |
| *asnB* | M-Z18AGL008364 | K01953 | Asparagine synthase (glutamine-hydrolysing) | -14.51 | -2.63 |
| *asnB* | M-Z18AGL008366 | K01953 | Asparagine synthase (glutamine-hydrolysing) | -12.03 | -3.7 |
| *mmuM* | M-Z18AGL001462 | K00547 | Homocysteine S-methyltransferase | 1.7 | 1.44 |
| *metH* | M-Z18AGL006552 | K00548 | 5-Methyltetrahydrofolate--homocysteine methyltransferase | -1.97 | -1.23 |
| *thrB* | M-Z18AGL000214 | K02204 | Homoserine kinase type II | -5.81 | 1.7 |
| *thrC* | M-Z18AGL003747 | K01733 | Threonine synthase | -1.18 | -1.32 |

1. * Corresponding author at: The Key Laboratory of Industrial Biotechnology, Ministry of Education, School of Biotechnology, Jiangnan University, 1800 Lihu Road, Wuxi 214122, Jiangsu, China

   *E-mail address*: [chenxs@jiangnan.edu.cn](mailto:chenxs@jiangnan.edu.cn) (X.S. Chen) [↑](#footnote-ref-1)
